# Lessons Learned on Obtaining Reliable Conductivity Estimates From Molecular Dynamics Simulations

**DOI:** 10.1002/cphc.70477

**Published:** 2026-07-24

**Authors:** Paul Zaby, Johannes Ingenmey, Tuanan C. Lourenço, Yong Zhang, Juarez L. F. Da Silva, Martin Brehm, Edward J. Maginn, Barbara Kirchner

**Affiliations:** ^1^ Mulliken Center for Theoretical Chemistry University of Bonn Bonn Germany; ^2^ Department of Physics and Mathematics Institute of Chemistry São Paulo State University Araraquara São Paulo Brazil; ^3^ Department of Chemical and Biomolecular Engineering College of Engineering University of Notre Dame Notre Dame Indiana USA; ^4^ São Carlos Institute of Chemistry University of São Paulo São Carlos São Paulo Brazil; ^5^ Department of Chemistry Paderborn University Paderborn Germany

**Keywords:** computational chemistry, ionic liquids, molecular dynamics

## Abstract

The calculation of reliable ionic conductivities from molecular dynamics simulations is not a straightforward task, especially for strongly correlated systems, such as ionic liquids or highly concentrated electrolytes, where the Nernst–Einstein approach tends to fail. In this manuscript, we present the newly implemented conduct module for TRAVIS. It allows the calculation of the ionic conductivity using the Einstein–Helfand and Green–Kubo approaches, which explicitly include ionic correlations in their formalism. We provide a broad overview of accessible transport properties and compare methods and best practices for obtaining statistically reliable estimates of ionic conductivity and other physicochemical properties derived from electrolyte molecular dynamics simulations, including transport numbers and the inverse Haven ratio. To validate our implementation and demonstrate the conduct module's capabilities, we simulated the ionic liquid 1‐ethyl‐3‐methylimidazolium dicyanamide ([EMIm][DCA]) as well as the ether‐based electrolyte lithium bis(fluorosulfonyl)imide in ethylene glycol dimethyl ether (LiFSI/DME).

## Graphical abstract

We present the newly implemented “conduct” module for TRAVIS. It allows the calculation of ionic conductivities from molecular dynamics simulations using the Einstein–Helfand and Green–Kubo approaches. We provide a broad overview of accessible transport properties and compare methods and best practices for obtaining statistically reliable estimates. To demonstrate the “conduct” module's capabilities, we simulated [EMIm][DCA] as well as LiFSI/DME.

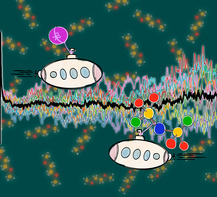

## 1 Introduction

Since the first trajectory studies on simple liquids [1], molecular dynamics (MD) simulations have become a standard tool for the investigation and prediction of the structural and dynamic properties of liquids [2]. General analysis packages, such as PyLat [3], MDSuite [4], TRAVIS [5, 6, 7], and others [8, 9, 10], play an important role in facilitating the consistent and reproducible extraction of structural [11, 12], spectroscopic [13, 14], and dynamic [15] information from simulations. A dynamic property of particular importance for electrolytes is the ionic conductivity. Early computational studies of the ionic conductivity were limited to systems like aqueous NaCl solutions [16], but over the past two decades, MD has been routinely used to investigate charge transport in ionic liquids [17, 18, 19, 20, 21, 22] and concentrated electrolytes [23, 24, 25]. Recent works have focused on the development of robust and uncertainty‐aware approaches for the computation of transport coefficients from simulation [9, 10, 26, 27, 28, 29, 30, 31, 32].

In the idealized picture of a dilute electrolyte, the ionic conductivity is the sum of single‐particle contributions by charged species. It is directly connected to their self‐diffusion coefficients by the Nernst–Einstein (NE) relation [33]. However, unlike self‐diffusion coefficients [34], the ionic conductivity is a collective transport property and may significantly deviate from the NE relation, especially in systems where ion–ion correlations become prominent [35, 36, 37, 38]. Computational investigations of the ionic conductivity in highly concentrated systems such as ionic liquids or water‐in‐salt electrolytes [39, 40] therefore require methods that can resolve these distinct microscopic contributions [41]. In equilibrium MD, the ionic conductivity can be obtained either from the charge current autocorrelation function (CACF) in the Green–Kubo (GK) formulation [42, 43] or from the long‐time growth of the collective mean squared displacement (CMSD) in the Einstein–Helfand (EH) representation [44].

Although the GK and EH approaches are formally equivalent, they are not always equally accessible in practice. The GK conductivity estimate is sensitive to the cutoff criterion used on the integral of the CACF. The long‐time tail of the CACF is noisy, complicating the localization of the plateau region in its integral. Some GK schemes address this problem through different uncertainty‐aware strategies including error‐controlled truncation [27], random‐walk modeling of tail noise [28], and estimation of the zero‐frequency limit of the power spectrum [10]. Recently, Otero‐Lema et al. published KUTE, an uncertainty‐weighted running integral estimator with plateau detection [9].

The EH approach requires identifying the linear diffusive regime in the CMSD. Choosing an appropriate fit window is often not trivial due to non‐diffusive behavior at short time scales and increasingly noisy long‐time data. Large variances in the CMSD introduced by cross‐correlations make single‐replica estimates unreliable, even when they appear sufficiently linear [45]. In recent works, an increased emphasis is placed on the importance of uncertainty quantification and eliminating user bias [26].

Obtaining reliable conductivity estimates is further complicated by the choice of model to represent the system of interest [46]. Explicitly polarizable force fields come with an increased computational demand but can significantly improve the dynamics of ionic systems. Recent studies have emphasized the importance of polarizability for the realistic modeling of ionic liquids [47, 48, 49]. Early works often used Madden‐type polarizable ion models for ionic liquids and salt melts [50, 51, 52]. Later, the polarizable modeling of ionic liquids was greatly facilitated by CL&Pol [53], a transferable and polarizable force field derived from the widely used fixed‐charge CL&P [54] force field. It was later extended to protic ionic liquids, deep eutectic solvents, and electrolytes [55]. At the same time, nonpolarizable force fields are still widely used, as they remain an attractive choice due to their accessibility and low computational cost [26]. However, fixed‐charge models tend to overestimate electrostatic interactions in concentrated ionic systems and ignore charge transfer effects [56], which slows down dynamics and distorts the computed transport properties [57]. A common strategy to account for this is to scale the atomic charges by a factor typically close to 0.8 [48], whereas more elaborate approaches derive an explicit scaling factor from the dielectric constant [58], from Voronoi integration [59] or other variants [60]. Furthermore, explicit re‐parametrization of force fields has shown enormous success in several instances [26, 61, 62, 63]. Beyond classical force‐field simulations, recent studies have increasingly utilized ab initio [64, 65] and machine learning potentials [66, 67] for conductivity calculations, including reactive aqueous electrolytes [68] in which proton transfer contributions to conductivity are essential.

Closely related to the ionic conductivity are transport numbers [69, 70], which measure how different ionic species contribute to the electrical current. Unlike the total conductivity, which is independent of the choice of reference frame, partial conductivities and the transport numbers derived from them are sensitive to the reference frame [35, 36, 70, 71, 72, 73, 74]. Quantities derived from MD simulation are typically measured in the mass‐fixed frame, whereas experimental transport numbers are usually reported for the solvent‐fixed frame. This distinction can significantly affect the transport numbers’ numeric value and even change their sign [70]. In ionic liquids, the absence of a neutral solvent complicates the interpretation of transport numbers, as there is no natural analog of the solvent‐fixed frame commonly used in experiments [35, 72, 73].

These considerations highlight the challenge of calculating ionic conductivity estimates from equilibrium MD simulations. Therefore, in this paper, we showcase the functionality of the conduct module, which has been newly implemented into TRAVIS and allows the calculation of ionic conductivities from MD trajectories using both the GK and EH approaches, as well as their decomposition into different ion‐resolved self‐ and cross‐term contributions. The module supports on‐the‐fly changes of reference frame, eliminating the need for preprocessing of the trajectory. In addition, we address recommended best practices for obtaining reliable and statistically significant conductivity estimates and related transport properties.

## 2 Methodology: Ionic Conductivity and Trajectory Analysis

### 2.1 Ionic Conductivity

In equilibrium MD simulations, the ionic conductivity can be estimated either from single‐particle motion using the NE approximation or from collective charge transport using formally exact approaches derived from linear response theory. The NE approximation neglects correlated ionic motion in the system and estimates the ionic conductivity as a sum of self‐contributions. In contrast, the EH and GK approaches include self‐ and cross‐correlation terms explicitly. EH determines the conductivity from the long‐time growth of the CMSD of the charged particles, whereas GK obtains it from the time integral of the CACF.

In the infinite‐time limit, the EH and GK methods are formally equivalent and yield identical values for the ionic conductivity. In practice, however, finite‐trajectory estimators can differ depending on sampling quality and the choice of fitting or plateau finding protocol, as well as their numeric requirements. The EH approach is especially helpful for systems with strong ionic correlations. Nevertheless, it requires sufficiently long trajectories in order to robustly determine regions in which the CMSD grows linearly (diffusive regimes). Additionally, the EH method relies on continuous, unwrapped ion coordinates in order to evaluate long‐time displacements correctly. The GK approach, in contrast, is based on microscopic charge currents and therefore does not require unwrapped coordinates, provided that reliable velocities of the charged species are available. However, these velocities need to be resolved on the femtosecond timescale, and sufficiently long correlation times must be sampled. Both approaches require a good statistical convergence of the respective trajectories. All abbreviations and variables introduced in this section are collected in Supporting Information Section S2.

#### 2.1.1 NE Conductivity From Self‐Diffusion Coefficients

The NE approximation estimates the ionic conductivity solely from the self‐diffusion coefficients *D*_k_ of all charged species *k* in the system, without accounting for the correlated movement of ions:

(1) $$\sigma_{\mathrm{NE}}=\frac{e^{2}}{Vk_{B}T}\sum_{k=1}^{C}z_{k}^{2}N_{k}D_{k}=\sum_{k=1}^{C}\sigma_{k}^{\mathrm{self}}$$

where *e* is the elementary charge, *V* the volume of the system, *T* the temperature, *k*_B_ the Boltzmann constant, and *C* the number of charged species in the system. Furthermore, *z*_k_ denotes the (dimensionless) signed valence of ionic species *k*, such that the physical charge is *q*_k_ = *ez*_k_, and *N*_k_ the number of ions of the respective species in the system. The self‐diffusion coefficients in Equation (1) can be obtained by either the Einstein relation, based on the mean squared displacement, or the GK relation, based on the velocity autocorrelation function (VACF); see Supporting Information Section S5 for details.

The NE approximation is well suited for dilute electrolyte solutions in which the correlated movement of ions is negligible. However, in liquids with high ion concentrations like ionic liquids or solvent‐in‐salt electrolytes, the NE conductivity can deviate significantly from the true conductivity.

#### 2.1.2 EH Conductivity From CMSD

In the EH approach, the ionic conductivity is obtained by the following equation:

(2) $$\begin{array}{c} \sigma_{\mathrm{tot}}^{\mathrm{EH}}=\frac{e^{2}}{6Vk_{B}T}\lim_{\tau \to \infty }\frac{\partial }{\partial \tau }{\mathrm{MSD}}^{\mathrm{col}}\left(\tau \right)\\ {\mathrm{MSD}}^{\mathrm{col}}\left(\tau \right)={\left\langle \sum_{i=1}^{N}\sum_{j=1}^{N}z_{i}z_{j}\cdot \Delta {\vec{r}}_{i}(t,\tau )\cdot \Delta {\vec{r}}_{j}(t,\tau )\right\rangle }_{t} \end{array}$$

where $\Delta {\vec{r}}_{i}(t,\tau )={\vec{r}}_{i}(t+\tau )-{\vec{r}}_{i}(t)$ is the displacement vector of ion *i* from time *t* to *t* + *τ* for a given lag time *τ*. The representation in Equation (2) suggests a naive O(*N* ^2^) scaling. However, using the following identity

$$\begin{array}{c} \sum_{i=1}^{N}\sum_{j=1}^{N}{\vec{v}}_{i}\cdot {\vec{v}}_{j}={\left\|\sum_{i=1}^{N}{\vec{v}}_{i}\right\|}_{2}^{2} \end{array}$$

where ${\left\|\cdot \right\|}_{2}$ denotes the Euclidean norm and $\vec{v}$ is an arbitrary vector, Equation (2) can be rewritten into the equivalent but more practical form

(3) $$\sigma_{\mathrm{tot}}^{\mathrm{EH}}=\frac{e^{2}}{6Vk_{B}T}\lim_{\tau \to \infty }\frac{\partial }{\partial \tau }{\left\langle {\left\|\sum_{i=1}^{N}z_{i}\Delta {\vec{r}}_{i}(t,\tau )\right\|}_{2}^{2}\right\rangle }_{t}$$

Since Equations (2) and (3) contain the full pairwise sum over all ions in the system, the total ionic conductivity can be decomposed into self‐terms (*i* = *j*) and cross‐terms (*i* ≠ *j*), which can subsequently be separated according to the species identities of the respective ions, such that for a system containing only two ionic species,

(4) $$\sigma_{\mathrm{tot}}^{\mathrm{EH}}=\sigma_{+}^{\mathrm{self}}+\sigma_{-}^{\mathrm{self}}+\sigma_{++}^{\mathrm{cross}}+\sigma_{--}^{\mathrm{cross}}+\sigma_{+-}^{\mathrm{cross}}$$

where $\sigma_{++}^{\mathrm{cross}}$, $\sigma_{--}^{\mathrm{cross}}$, and $\sigma_{+-}^{\mathrm{cross}}$ are the contributions to the conductivity arising from the correlated movement of like‐charged and oppositely charged ions. Note that, in our notation, the cross‐terms include each mixed (*i*,*j*) contribution twice, due to the symmetry of the double sum in Equation (2). Additionally, the species‐related collective contributions (here for the case of the cation) can be defined as

(5) $$\sigma_{+}^{\mathrm{EH}}=\sigma_{+}^{\mathrm{self}}\;+\;\sigma_{++}^{\mathrm{cross}}=\frac{e^{2}}{6Vk_{B}T}\lim_{\tau \to \infty }\frac{\partial }{\partial \tau }{\left\langle {\left\|\sum_{i=1}^{N_{+}}z_{i}\Delta {\vec{r}}_{i}(t,\tau )\right\|}_{2}^{2}\right\rangle }_{t}$$

It can be easily seen from Equation (5) that species‐related collective contributions can be obtained in linear time (regarding the number of ions). Since also the self‐contributions $\sigma_{+}^{\mathrm{self}}$ can be obtained in linear time via the self‐diffusion coefficients, the same‐species cross‐correlation terms, i.e., $\sigma_{++}^{\mathrm{cross}}$, are straightforwardly obtained as well. The remaining cross‐species correlation terms, such as $\sigma_{+-}^{\mathrm{cross}}$, result from subtracting the species‐related collective contributions from the total conductivity.

Assuming more than two ionic species, the total ionic conductivity can be written as

(6) $$\sigma_{\mathrm{tot}}^{\mathrm{EH}}=\sum_{k=1}^{C}\sigma_{k}^{\mathrm{EH}}+\sum_{k<l}\sigma_{\mathrm{kl}}^{\mathrm{cross}}$$

In order to obtain the cross‐terms in linear time, additional pair contribution terms need to be defined. Thus, for a pair of species (k, l), the pair contribution is

(7) $$\sigma_{\mathrm{kl}}^{\mathrm{EH}}=\sigma_{k}^{\mathrm{EH}}+\sigma_{l}^{\mathrm{EH}}+\sigma_{\mathrm{kl}}^{\mathrm{cross}}=\frac{e^{2}}{6Vk_{B}T}\lim_{\tau \to \infty }\frac{\partial }{\partial \tau }{\left\langle {\left\|\sum_{i\in \{k,l\}}z_{i}\Delta {\vec{r}}_{i}(t,\tau )\right\|}_{2}^{2}\right\rangle }_{t}$$

which makes it possible to obtain the inter‐species cross‐term $\sigma_{\mathrm{kl}}^{\mathrm{cross}}$ as

(8) $$\sigma_{\mathrm{kl}}^{\mathrm{cross}}=\sigma_{\mathrm{kl}}^{\mathrm{EH}}-\sigma_{k}^{\mathrm{EH}}-\sigma_{l}^{\mathrm{EH}}$$

For any number of ionic species in a system, every single self‐ and cross‐component can therefore be obtained in linear time with respect to the number of ions *N*. The total time complexity of the entire decomposition therefore lies in O(*C* ^2^ *NT*_max_), where *C* is the number of ionic species, *N* is the total number of ions, and *T*_max_ is the number of trajectory frames used to compute the collective MSDs.

#### 2.1.3 GK Conductivity From CACF

An alternative approach to obtain the ionic conductivity from MD trajectories is the GK approach. Here, the CACF is integrated over lag time:

(9) $$\sigma_{\mathrm{tot}}^{\mathrm{GK}}=\frac{e^{2}}{3k_{B}TV}\int_{0}^{\infty }{\left\langle \vec{J}(t+\tau )\cdot \vec{J}(t)\right\rangle }_{t}\;d\tau ,\;\vec{J}(t)=\sum_{i=1}^{N}z_{i}{\vec{v}}_{i}(t)$$

where $\vec{J}(t)$ denotes the (reduced) microscopic charge current defined in terms of the dimensionless valences *z*_i_ and ${\vec{v}}_{i}$ is the (center‐of‐mass) velocity of ion *i*. The correlation function can be obtained efficiently using the fast Fourier transform (FFT).

Similar to the EH ansatz, the total conductivity can be decomposed into self‐ and cross‐terms. As already mentioned above, the self‐term (proportional to the self‐diffusion coefficient) of an ionic species *k* can be obtained as a sum of VACFs of single ions *i*:

(10) $$\sigma_{k}^{\mathrm{self},\mathrm{GK}}=\frac{e^{2}z_{k}^{2}}{3k_{B}TV}\sum_{i=1}^{N_{k}}\int_{0}^{\infty }{\left\langle {\vec{v}}_{i}(t+\tau )\cdot {\vec{v}}_{i}(t)\right\rangle }_{t}\;d\tau$$

By defining species currents, as well as pair currents

(11) $${\vec{J}}_{k}(t)=\sum_{i=1}^{N_{k}}z_{i}{\vec{v}}_{i}(t),\;{\vec{J}}_{\mathrm{kl}}(t)={\vec{J}}_{k}(t)+{\vec{J}}_{l}(t)$$

one can obtain the species‐related contributions in a GK relation as

(12) $$\sigma_{k}^{\mathrm{GK}}=\frac{e^{2}}{3k_{B}TV}\int_{0}^{\infty }{\left\langle {\vec{J}}_{k}(t+\tau )\cdot {\vec{J}}_{k}(t)\right\rangle }_{t}\;d\tau$$

and for pair contributions:

(13) $$\sigma_{\mathrm{kl}}^{\mathrm{GK}}=\frac{e^{2}}{3k_{B}TV}\int_{0}^{\infty }{\left\langle {\vec{J}}_{\mathrm{kl}}(t+\tau )\cdot {\vec{J}}_{\mathrm{kl}}(t)\right\rangle }_{t}\;d\tau$$

Thus, analogous to EH, all self‐ and cross‐terms that make up the total conductivity can be obtained with a limited amount of correlation functions linear in the number of ions *N*.

#### 2.1.4 Derived Quantities

Transport numbers, *t*_k_, often called transference numbers, are used to measure the fraction of charge transported by a specific electrolyte species, *k*, in the system. As in the case of ionic conductivity, *t*_k_ can be obtained either from the self‐contributions alone or from the full conductivity decomposition. The former neglects ionic correlations, whereas the latter explicitly incorporates correlated movement of the ions by the *σ*_k_ and $\sigma_{\mathrm{kl}}^{\mathrm{cross}}$ terms obtained from the EH or GK approach. When only the self‐contribution terms are used, the transport numbers are often called “ideal” transport numbers, $t_{k}^{\mathrm{id}}$, and can be obtained by the following equation:

(14) $$t_{k}^{\mathrm{id}}=\frac{z_{k}^{2}\cdot N_{k}\cdot D_{k}}{\sum_{l=1}^{C}z_{l}^{2}N_{l}D_{l}}=\frac{\sigma_{k}^{\mathrm{self}}}{\sigma_{\mathrm{NE}}}$$

On the other hand, the transport numbers calculated using all the *σ*_k_ and *σ*_kl_ terms, i.e., taking into account the correlated motion in the system, are often called “real” transport numbers, $t_{k}^{\mathrm{real}}$, and can be obtained by the following equation,

(15) $$t_{k}^{\mathrm{real}}=\frac{\sigma_{k}+\frac{1}{2}\sum_{k\neq l}\sigma_{\mathrm{kl}}^{\mathrm{cross}}}{\sigma_{\mathrm{tot}}}$$

where *σ*_k_ is the collective contribution related to species *k* (as defined in Equation (5)), and the inter‐species cross‐terms $\sigma_{\mathrm{kl}}^{\mathrm{cross}}$ are distributed equally between the two species *k* and *l*. These transport numbers can be calculated using the decomposition of either the EH or GK approach. However, they are reference frame dependent, which can impose challenges when comparing theoretical data from MD simulations to experimental measurements (see the reference frame discussion below).

As a measure for the deviation from ideal, uncorrelated ion transport, the inverse Haven ratio can be used:

(16) $$H^{-1}=\frac{\sigma_{\mathrm{tot}}}{\sigma_{\mathrm{NE}}}$$

### 2.2 Reference Frames

In finite systems, quantities like charge currents and cross‐correlations can depend on the frame of reference that is used to define position as well as velocity vectors [36, 70, 71]. Here, we focus on three reference frames that are common in literature: the mass‐fixed (barycentric), the number‐fixed (stoichiometric), and the solvent‐fixed frame of reference [70]. All of these frames are available in the conduct module in travis.

Starting from arbitrary particle velocities ${\vec{v}}_{i}(t)$ (ions or neutral molecules), the transformation into any reference frame *α* yields new velocities ${\vec{v}}_{i}^{\left(\alpha \right)}(t)$:

(17) $${\vec{v}}_{i}^{\left(\alpha \right)}(t)={\vec{v}}_{i}(t)-{\vec{w}}^{\left(\alpha \right)}(t)$$

where ${\vec{w}}^{\left(\alpha \right)}(t)$ is the reference velocity in the respective reference frame. For the barycentric frame, this velocity ${\vec{w}}^{\left(M\right)}(t)$ is the center‐of‐mass velocity:

(18) $${\vec{w}}^{\left(M\right)}(t)=\frac{1}{M}\sum_{i=1}^{N_{\mathrm{tot}}}m_{i}\;{\vec{v}}_{i}(t)$$

where *m*_i_ is the mass of particle *i* and *M* is the total mass of the system consisting of *N*_tot_ particles (including both ions and neutral molecules). Analogously, the number‐fixed frame employs the arithmetic mean of the center‐of‐mass velocities of all particles in the system as reference, whereas for the solvent‐fixed frame, only the *N*_solv_ solvent molecules are taken into account:

(19) $$\begin{array}{c} {\vec{w}}^{\left(N\right)}(t)=\frac{1}{N_{\mathrm{tot}}}\sum_{i=1}^{N_{\mathrm{tot}}}{\vec{v}}_{i}(t) \end{array}$$

(20) $$\begin{array}{c} {\vec{w}}^{\left(S\right)}(t)=\frac{1}{N_{\mathrm{solv}}}\sum_{i=1}^{N_{\mathrm{solv}}}{\vec{v}}_{i}(t) \end{array}$$

Most contributions to the ionic conductivity depend strongly on the reference frame; however, the total conductivity and the self‐terms of the ionic species are independent of the reference frame employed (for systems that are charge‐neutral and are close to the thermodynamic limit) [70]. This will be shown in the following.

#### 2.2.1 Reference Frame Independence of Total Conductivity

For both the EH and GK formulations, reference frame independence follows directly from the overall charge neutrality of the system (i.e., $\sum_{i=1}^{N}z_{i}=0$) [71]. In order to show this for the EH approach, the sum in Equation (3) must be addressed. Assuming that the origin of the chosen reference frame *α* is represented by the vector ${\vec{R}}^{\left(\alpha \right)}(t)$ in our initial coordinate system, the displacement vector $\Delta {\vec{r}}_{i}(t,\tau )$ of the ion *i* transforms as

(21) $$\Delta {\vec{r}}_{i}^{\left(\alpha \right)}(t,\tau )=\Delta {\vec{r}}_{i}(t,\tau )-\Delta {\vec{R}}^{\left(\alpha \right)}(t,\tau )$$

Considering the summation over all ions, one can see that the collective MSD stays invariant:

(22) $$\sum_{i=1}^{N}z_{i}\Delta {\vec{r}}_{i}^{\left(\alpha \right)}(t,\tau )=\sum_{i=1}^{N}z_{i}\left(\Delta {\vec{r}}_{i}(t,\tau )-\Delta {\vec{R}}^{\left(\alpha \right)}(t,\tau )\right)=\sum_{i=1}^{N}z_{i}\Delta {\vec{r}}_{i}(t,\tau )-\Delta {\vec{R}}^{\left(\alpha \right)}(t,\tau )\underset{=\;0}{\underbrace{\left(\sum_{i=1}^{N}z_{i}\right)}}=\sum_{i=1}^{N}z_{i}\Delta {\vec{r}}_{i}(t,\tau )$$

Similarly, applying any frame transformation to the total charge current $\vec{J}(t)$ reveals the independence of the total GK conductivity:

(23) $${\vec{J}}^{\left(\alpha \right)}(t)=\sum_{i=1}^{N}z_{i}\left({\vec{v}}_{i}(t)-{\vec{w}}^{\left(\alpha \right)}(t)\right)=\sum_{i=1}^{N}z_{i}{\vec{v}}_{i}(t)-{\vec{w}}^{\left(\alpha \right)}(t)\underset{=\;0}{\underbrace{\left(\sum_{i=1}^{N}z_{i}\right)}}=\vec{J}(t)$$

Thus, no matter how it is obtained, the total conductivity is independent of the frame of reference.

#### 2.2.2 Self‐Contributions in the Thermodynamic Limit

The reference frame dependence of the self‐diffusion coefficients is a finite size effect. Under any global frame transformation, according to Equation (21), the mean squared displacement transforms as

(24) $$\begin{array}{c} {\mathrm{MSD}}_{k}^{\left(\alpha \right)}(\tau )={\mathrm{MSD}}_{k}(\tau )-2{\left\langle \Delta {\vec{r}}_{i}(t,\tau )\cdot \Delta {\vec{R}}^{\left(\alpha \right)}(t,\tau )\right\rangle }_{t,i}\\ \;+{\left\langle {\left\|\Delta {\vec{R}}^{\left(\alpha \right)}(t,\tau )\right\|}_{2}^{2}\right\rangle }_{t} \end{array}$$

The additional terms describe the correlations of single ions with the movement of the reference point (i.e., the center of mass). Since typically the reference point results from the average of many particles, every single particle contributes only weakly to the reference (approximately O(1/*N*)). Additionally, the fluctuations in the squared norm reference vector normally are in the order of O(1/*N*) as well. Thus, for large systems (large *N*), these additional terms are negligible, and

$${\mathrm{MSD}}_{k}^{\left(\alpha \right)}\left(\tau \right)\underset{N\to \infty }{\to }{\mathrm{MSD}}_{k}(\tau )$$

Analogously, the self‐terms in the GK approach (see Equation (10)) are independent of the choice of reference frame in the thermodynamic limit. Applying a frame transformation to Equation (10) using the reference velocity ${\vec{w}}^{\left(\alpha \right)}(t)$, similar additional terms appear in the VACF. The respective correlations with ${\vec{w}}^{\left(\alpha \right)}(t)$ are negligible for the same reasons as above, if the number of particles that define the reference velocity is large (as is normally the case in mass‐fixed, number‐fixed, and solvent‐fixed reference frames for MD simulations).

In total, one arrives at the conclusion that the only contributions to the ionic conductivity that are not frame independent (even in the thermodynamic limit) are the separate cross‐terms [71]. These properties of the different ionic contributions need to be considered when calculating and interpreting the decomposition of conductivities and derived quantities such as transport numbers.

### 2.3 Analysis Techniques

In this section, the numerical evaluation of GK and EH quantities from a set of MD trajectories, as well as the strategies for determining robust long‐time limits (plateaus and linear regimes) and related uncertainties, is described. Since TRAVIS is optimized to work with single trajectories, all analyses of the individual trajectories in common formats such as .lmp, .gro, and .xyz are performed with TRAVIS. For analyses that require input from several replicas, we wrote simple Python scripts which are available in our Github repository (see the Code Availability Statement). All of the described methods can also be applied to any contributions to the total conductivity (self‐ or cross‐terms) or their linear combinations.

#### 2.3.1 EH—Determination of Slope

In the EH approach, the collective MSD is calculated for every trajectory available, and from the long‐time regime, the conductivity can be obtained using linear regression. Therefore, it is necessary to choose a suitable linear regime to which the fit should be applied. Within the conduct module of TRAVIS, identification of such a regime is done automatically, based on the logarithmic slope of the collective MSD (as already presented by Frömbgen et al.) [26], yielding an algorithmically determined estimate of the linear regime. In summary, the effective slope $d\log\;{\mathrm{MSD}}^{\mathrm{col}}/d\log\;\tau$ is evaluated in sliding windows, and intervals with slopes close to one are classified as diffusive or linear regimes. The computed slope is then comparable to an effective exponent evaluation. Additionally, a minimal interval length is required in order to obtain stable regression results. Therefore, all trajectories used for the EH analysis below are analyzed up to a correlation depth of 30 ns, corresponding to 30% of the total trajectory length.

**FIGURE 1:**
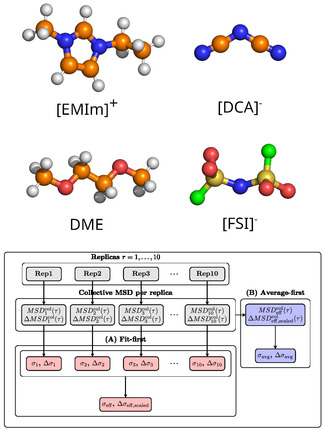
Top: Molecular representations for 1‐ethyl‐3‐methylimidazolium, [EMIm]^+^; dicyanamide, [DCA]^−^; ethylene glycol dimethyl ether, DME; and bis(fluorosulfonyl)imide, [FSI]^−^. Atoms H, C, N, O, F, and S are colored white, orange, blue, red, green, and yellow, respectively. Bottom: Workflow for the Einstein–Helfand approach. Gray nodes indicate obligatory work steps, red nodes indicate steps necessary for EH approach (A), and blue nodes correspond to the steps needed for EH approach (B).

Two complementary methods (illustrated in Figure 1 bottom) can be used:

*Error‐Weighted Fit per Trajectory; Then Weighted Average*

For every trajectory *r*, the respective conductivity *σ_r_* is computed by TRAVIS via weighted linear regression within the automatically determined linear regime. The respective weights are the inverse of the squared standard errors $\Delta {\mathrm{MSD}}_{r}^{\mathrm{col}}\left(\tau \right)$, estimated from the fluctuations of ${\mathrm{MSD}}_{r}^{\mathrm{col}}\left(\tau \right)$ over different time origins. The resulting conductivities of the separate replicas *σ_r_* are attributed uncertainties [31] equal to the fit errors Δ*σ_r_*.

Utilizing the results printed by TRAVIS, the effective conductivity *σ*_eff_ can be obtained as weighted average over the replica conductivities *σ*_*r*_, assuming the different replicas are statistically independent (i.e., generated from distinct initial configurations and/or velocity seeds):

(25) $$\sigma_{\mathrm{eff}}=\frac{\sum_{r}w_{r}\sigma_{r}}{\sum_{r}w_{r}},\;w_{r}=\frac{1}{{\left(\Delta \sigma_{r}\right)}^{2}}$$

The corresponding standard error is given by

(26) $$\Delta \sigma_{\mathrm{eff}}=\sqrt{\frac{1}{\sum_{r}w_{r}}}$$

In order to take inconsistencies between scatter and replica uncertainties into account, a reduced chi‐squared statistic can be used:

(27) $$\begin{array}{c} \Delta \sigma_{\mathrm{eff},\mathrm{scaled}}=\Delta \sigma_{\mathrm{eff}}\sqrt{\chi_{\mathrm{red}}^{2}}\\ \chi_{\mathrm{red}}^{2}=\frac{\sum_{r}w_{r}{\left(\sigma_{r}-\sigma_{\mathrm{eff}}\right)}^{2}}{N_{r}-1} \end{array}$$

If the observed scatter between replicas turns out to be larger than suggested by the separate Δ*σ_r_*, the factor $\sqrt{\chi_{\mathrm{red}}^{2}}$ can increase the uncertainty accordingly. This method is able to more reliably capture the scatter between replicas but requires good sampling within the separate replicas already.

*Weighted Average Over Collective MSD; Then Error‐Weighted Fit*

Alternatively, since TRAVIS provides detailed records of the raw data, ${\mathrm{MSD}}_{r}^{\mathrm{col}}\left(\tau \right)$ can be averaged directly over the different replicas *r*. Here, the weights are the inverse variances of each data point (*τ*) within the collective MSDs. The weighted average ${\mathrm{MSD}}_{\mathrm{eff}}^{\mathrm{col}}\left(\tau \right)$ as well as its uncertainty can be obtained according to Equations (25) and (27). Afterward, a linear regime can be detected on ${\mathrm{MSD}}_{\mathrm{eff}}^{\mathrm{col}}\left(\tau \right)$ (here, by employing MSDiff version 0.2.0 [26]), which is then fitted using weighted regression. This method is especially superior, if the collective MSDs of the single replicas show large fluctuations/noise, and thus, identification of a reasonably linear regime is not straightforward.

It is possible to improve both methods using bootstrapping. Therefore, replicas need to be sampled with replacement, and for every bootstrap sample, an effective conductivity is calculated. The distribution of effective conductivities then yields reliable estimates of conductivity and uncertainty.

#### 2.3.2 GK—Determination of Integral

In the GK approach, first, the charge current $\vec{J}(t)$ is calculated for every saved trajectory frame. From this, the autocorrelation function $\left\langle \vec{J}(t)\cdot \vec{J}(0)\right\rangle$ can be efficiently evaluated using FFT. The integral of the correlation function over time results in a time‐dependent integral curve. In order to integrate the correlation function numerically with reasonable accuracy, a high dumping frequency of the velocities/charge current in the low femtosecond range is required. The largest dumping interval that can be used without losing important information is system dependent. Therefore, here, all trajectories analyzed with the GK approach were simulated with a dumping frequency of 1 fs to be certain that no significant contributions were missed. For the sake of memory management and to reduce bias in the statistical comparison of the two methods, all trajectories produced were limited to the same number of saved frames but differed in the dump frequency and total simulation time. The trajectories produced for analysis with the GK approach were run for 100 ps and processed up to a correlation depth of 75 ps.

In order to obtain the ionic conductivity, a region in which the integral converges against a constant value must be identified. For the single‐replica case, TRAVIS assumes the last third of the running integral to be fluctuating about the constant plateau value. Under this assumption, TRAVIS chooses the average of the integral values in this region as the estimated plateau value. However, for large times, the correlation function shows significant noise, making the integral fluctuate strongly without clear convergence (“long‐time noise problem,” see Figure 5). Therefore, it is necessary to assess the uncertainty of this simple estimator accurately, which is done by expressing the integral values in terms of finite differences and then employing the auto‐covariance of the latter (see Supporting Information Section S6 for details).

For averages over several replicas, the typical long‐time drifts of the integral can be addressed using an automatic plateau detection that tries to identify a region in which the averaged running integral shows no significant drift, yielding an algorithmically defined estimator of the plateau region and the corresponding conductivity.

The automatic plateau detection works analogously to the linear slope detection of MSDiff [26] and is performed with a small Python script. First, the integral curve is partitioned into (nslice) intervals, which are evaluated from long to short times. A candidate interval *I* is considered a plateau, if the absolute slope |d*I*/d*τ*| is below a given tolerance tol. Such plateaus are then iteratively expanded to shorter times with a step size of incr as long as the slope stays within the given tolerance. Among all identified plateaus, the candidate with the largest number of data points is chosen. If tied, the interval with the smaller absolute slope is preferred. In case no plateau can be found in the whole integral curve, the tolerance tol is increased stepwise and the process is started anew. This procedure is repeated until a plateau is found. In order to avoid using the short‐time correlation region by accident, the first interval of the integral curve is discarded. From the chosen plateau, the mean of the integral values in this region is then used as the conductivity value.

Additionally, for more than one independent replica, the mean and uncertainty can also be obtained by bootstrapping. Therefore, the replicas are sampled with replacement, and for every bootstrap sample, the correlation functions are averaged over all replicas in the respective sample. The resulting averages are then integrated, and the plateau is automatically detected. The scattering of the resulting plateau values yields the mean and the standard deviation of the mean distribution, which are reliable estimates for conductivity and uncertainty.

The single‐replica method should only be used if there is not more than one replica available or to obtain a crude estimate of the conductivity from the respective trajectory. Both of the multiple‐replica methods require a reliable identification of a suitable correlation depth interval, from which the asymptotic transport properties can be extracted. Note that there exist many alternative approaches to estimating the plateau value, such as the Cesàro summation [36, 37], or the uncertainty‐weighted average of the running integral implemented in the KUTE package [9].

## 3 Results and Discussion

To validate and demonstrate the capabilities of the conduct module in TRAVIS as well as to discuss our recommendations for ionic conductivity calculations, we performed classical MD for two representative systems (see Figure 1 top). The first system is the ionic liquid 1‐ethyl‐3‐methylimidazolium dicyanamide, [EMIm][DCA], for which simulations were carried out at different system sizes utilizing both a polarizable (CL&Pol) [53, 75] and a nonpolarizable (CL&P) [54] force field. System labels are written as IL^*N*^, where the superscript *N* denotes the number of ion pairs (e.g., IL^500^ refers to a system containing 500 ion pairs). Subscripts are used only to indicate either polarization (subscript “pol”) or increased simulation time (subscript “long”). The second system is the salt‐in‐solvent electrolyte lithium bis(fluorosulfonyl)imide in monoglyme, LiFSI/DME, investigated at three salt concentrations (1.0, 2.0, and 3.5 M), using the polarizable force field. For improved statistics, 10 independent replica simulations were performed for each set of conditions. Computational details and system compositions are provided in Supporting Information Section S3. Note that some of the variables required to obtain reliable ionic conductivities, such as the number of replicas, system size, simulation length, and the correlation depths, may depend on the simulated system and its thermodynamical state.

The articles that are cited in the Supporting Information are referenced here as well to prevent under‐citation [76, 77, 78, 79, 80, 81, 82, 83, 84, 85, 86, 87, 88, 89, 90, 91, 92, 93, 94, 95, 96].

### 3.1 Transport Properties From Self‐ and Collective Dynamics

In this subsection, the different evaluation approaches are first applied to two representative electrolyte model systems, the ionic liquid [EMIm][DCA] and the salt‐in‐solvent electrolyte, LiFSI/DME. The aim is to provide a compact overview of the basic transport properties of the systems and not yet a detailed assessment of the individual evaluation protocols. First, the self‐diffusion coefficients are considered as a simple dynamic quantity. Afterward, the related conductivities and the derived quantities will be discussed.

#### 3.1.1 Self‐Diffusion Coefficients

By applying linear fits to the linear regimes of the mean squared displacements [26], followed by averaging over independent replicas using the same weighted average procedure described for the conductivities, the self‐diffusion coefficients are obtained and reported in Tables 1 and 2 (a corresponding bar graph is shown in Figure S9). The standard errors reported in these tables were computed from the uncertainty of the linear regression and propagated through the weighted averaging over replicas. Already at this level, we can gain insights into the ion mobility within the different systems. For the ionic liquid system, the diffusion coefficients of cation and anion are in the same order of magnitude (10^−10^ m^2 ^s^−1^), which is consistent with values typically observed for ionic liquids [53]. In all simulations, the anion shows larger values than the cation, which can be attributed to the smaller radius of the [DCA]^−^ ion compared to the [EMIm]^+^ cation and the corresponding higher mobility [22]. At the same time, a systematic increase in the diffusion coefficient is observed within the CL&P series with increasing system size, indicating remaining finite size effects in the dynamic observables [97]. Comparing the IL^1000^ and ${\mathrm{IL}}_{\mathrm{pol}}^{1000}$ simulations, it can be seen that both ions exhibit increased mobility in the polarizable model. This is consistent with the frequently observed dynamic slowdown in nonpolarizable force‐field simulations of ionic liquids [53]. The shown standard errors are small compared to the observed differences between system sizes and force‐field variants, thus allowing for a robust identification of systematic trends. Additionally, the uncertainties within the CL&P series tend to decrease with increasing system size due to improved statistical sampling, even though the self‐diffusion coefficients themselves increase slightly. The ${\mathrm{IL}}_{\mathrm{long}}^{125}$ simulation, which is similar to the IL^125^ simulation but was run for a longer time of 800 ns, while only each 4000th timestep was written to the trajectory, also shows smaller errors than IL^125^, which again underscores the influence of available statistics on the precision of the determined quantities.

**TABLE 1:** Diffusion coefficients *D* and corresponding standard errors (SE) in 10^−10^ m^2^ s^−1^ for all simulations of [EMIm][DCA].

Simulation	Species	*D*	SE(*D*)
IL^125^	Anion	1.8807	0.0232
${\mathrm{IL}}_{\mathrm{long}}^{125}$		1.9158	0.0185
IL^250^		1.9755	0.0193
IL^500^		2.0398	0.0140
IL^1000^		2.0452	0.0124
${\mathrm{IL}}_{\mathrm{pol}}^{1000}$		2.4142	0.0143
IL^125^	Cation	1.61003	0.02184
${\mathrm{IL}}_{\mathrm{long}}^{125}$		1.62974	0.01519
IL^250^		1.67238	0.01312
IL^500^		1.72550	0.01085
IL^1000^		1.77472	0.00693
${\mathrm{IL}}_{\mathrm{pol}}^{1000}$		2.14962	0.00994

**TABLE 2:** Diffusion coefficients *D* and corresponding standard errors (SE) in 10^−10^ m^2^ s^−1^ for all simulations of the LiFSI/DME system.

Simulation	Species	*D*	SE(*D*)
1.0 M	Anion	5.5465	0.0779
2.0 M		2.0822	0.0214
3.5 M		0.3030	0.0060
1.0 M	Cation	5.0220	0.0667
2.0 M		2.1117	0.0140
3.5 M		0.3503	0.0043
1.0 M	Solvent	10.4773	0.0449
2.0 M		4.5053	0.0109
3.5 M		0.9117	0.0134

For the salt‐in‐solvent system, an even more pronounced trend can be observed; namely, with increasing salt concentration, the self‐diffusion coefficients of all species decrease substantially. This reduced mobility can be attributed to increased density, higher viscosity, and stronger structural coupling in the concentrated electrolyte systems [96]. Simultaneously, the solvent remains the most mobile species across all concentrations, reflecting that the neutral molecule is less affected by the electrostatic interactions present in the system. At lower concentrations, the anion is more mobile than the cation, but this difference decreases with increasing concentration and even slightly reverses at the high salt concentration of 3.5 M. This suggests that the relative ion mobilities in this system are not solely determined by mass and size of the ions but are increasingly influenced by concentration‐dependent solvation and ion association effects [96]. The reported absolute standard errors remain small in the salt‐in‐solvent system, also indicating a slight concentration‐dependent sampling effect.

Here, the diffusion coefficients primarily serve as reference quantities for the following analyses of ionic conductivities. Yeh–Hummer [88] correction terms were estimated and are reported in Supporting Information Section S5, Tables S4 and S5. Note that experimental viscosity data for the simulated systems are sparse and the calculated correction terms should be treated as qualitative approximations. Additionally, we want to stay consistent with the ionic conductivities reported in the next part, for which no similar corrections exist. For these reasons, we retain the raw diffusion coefficients for all comparisons and report the Yeh–Hummer corrections only for completeness.

#### 3.1.2 Conductivities and NE Approximation

**FIGURE 2:**
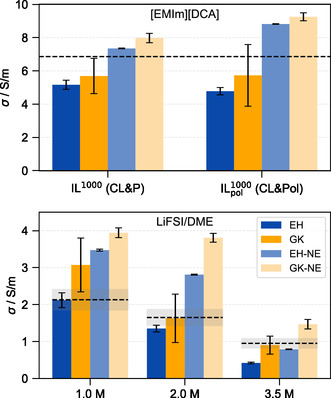
Conductivities for (top) [EMIm][DCA] computed from the IL^1000^ and ${\mathrm{IL}}_{\mathrm{pol}}^{1000}$ simulations and for (bottom) LiFSI/DME at different concentrations, obtained with the Einstein–Helfand (EH) and Green–Kubo (GK) approaches. For both approaches, the corresponding Nernst–Einstein conductivities (EH‐NE and GK‐NE) are shown as well. Experimental reference values are shown as dashed lines and were taken from (top) ref. [98] and interpolated from (bottom) refs. [86, 87], respectively; see Section S4.

Figure 2 compares the calculated EH, GK, and NE conductivities for both investigated systems. Whereas the NE approximation is solely based on the self‐diffusivities and thus does not incorporate ion–ion correlations, EH and GK capture the collective charge transport directly. Comparing these different approaches can therefore help to identify the importance of correlated movement of the ions in both model systems.

For the ionic liquid simulations with 1000 ion pairs, Figure 2, the EH and GK approaches yield a consistent qualitative trend, with the EH conductivities being systematically lower than the corresponding GK values for both force fields. The EH‐derived conductivities lie around 5.0 S m^−1^, while the corresponding GK values are approximately 0.7 S m^−1^ larger. This discrepancy can be attributed to the different types of trajectories used for the two analyses. Due to the large correlation depths needed to fit the collective MSD, the EH methods were applied to the 10 replicas of 100 ns simulations and a maximum correlation depth of 30 ns, while for the GK analysis, 10 trajectories of only 0.1 ns length were used, with velocities saved every 1.0 fs. Therefore, both approaches used the same number of trajectory frames, but on different timescales. This results in the final analyses being performed in different correlation depth regimes, leading to a small positive bias of the GK results compared to the EH results. To demonstrate the asymptotic equivalence of the EH and GK methods, additional analyses of the short trajectories and a single large trajectory are shown in Section S7.10. Even though they do not quantitatively agree, both collective approaches yield the same qualitative trend. However, both still deviate significantly from the reference value of 6.86 S m^−1^ obtained from experiment [98]. Note that this deviation does not imply an inadequacy of the employed analysis techniques but can be attributed to the inherent limitations of the underlying force fields and the inaccuracy of the reference data.

More pronounced than the differences in the mean values are the differences in uncertainties for EH and GK. Even though the trajectories consist of the same number of saved frames, the GK method presents a noisier pattern, which is due to the difficulty in identifying the plateau of the time‐integrated CACF. This is reflected in the shown standard errors, which are significantly larger for the GK conductivities, for both force fields.

Although the IL^1000^ and ${\mathrm{IL}}_{\mathrm{pol}}^{1000}$ simulations show substantial differences in the self‐diffusion coefficients (see Table 1), the collective conductivities only differ marginally. While the polarizable CL&Pol model predicts significantly higher diffusion coefficients, which is also reflected in the increased NE conductivities, this is not reflected in the EH and GK conductivities. The increased single‐particle mobilities therefore do not translate into increased macroscopic charge transport but are compensated by stronger ion–ion correlation effects.

Direct comparison of the collective conductivities with their NE counterparts further emphasizes the relevance of correlated movement of ions. For the EH and the GK approaches, the approximate uncorrelated conductivities (EH‐NE and GK‐NE) exceed the collective EH and GK conductivities significantly. Independent of the analysis protocol, the same qualitative observation is made: The NE approximation overestimates the effective charge transport due to missing ion–ion correlation effects [22]. Especially for ionic liquids, this is plausible due to the strong Coulomb interactions, local structuring, and coordinated ion movement typically observed in these systems.

At the same time, the simplified NE methods yield lower uncertainties compared to the collective approaches. This is not surprising, since the self‐contributions to the transport properties are generally better converged and less affected by statistical noise [22]. The numerical challenge lies therefore in the robust estimation of the contributions containing the collective movement of ions. For both force fields, the obtained GK‐NE values are slightly higher than those obtained from the EH‐NE approach, although these differences are of the same order of magnitude as the statistical uncertainties and can again be attributed to the different correlation depth regimes in which the analyses were performed. The EH and GK methods yield the same trends, despite differing in numerical stability.

In the salt‐in‐solvent system, we can investigate the observations made above in a concentration‐dependent manner. Overall, the values of all calculated conductivities decrease with salt concentration, which is consistent with the significant decrease in diffusive motion discussed earlier. Especially, the EH conductivities are able to accurately reproduce the experimentally observed concentration trend, with good agreement for 1.0 and 2.0 M concentrations. For the concentration 3.5 M, the experimental conductivity is underestimated, although the relative concentration trend is still well captured. Given the uncertainties of the simulation data and those of the reference data, the agreement is satisfactory. Although the GK conductivities again systematically exceed the EH values and carry significantly larger standard errors (for the same reasons as above, see Section S7.10), they still show the same overall trends. Nevertheless, the reference data also appear to be consistent with the GK results, when taking the large error bars into account.

For the collective approaches, the standard errors also follow a clear concentration trend. The uncertainties decrease with increasing salt concentration, which can primarily be attributed to improved sampling due to the larger number of ions in the high‐concentration systems. Since a significant part of the estimated uncertainty originates from the differences between replica simulations, a higher number of ions can help mitigate that by allowing for more efficient sampling of phase space within a single trajectory, thereby reducing variance between independent replicas. For the EH formulation, even the relative uncertainty (error divided by mean value) decreases with increasing concentration, highlighting the robustness of the method again. The NE approximation consistently yields overall smaller uncertainties, with only a weak concentration dependence in EH‐NE and essentially no trend in GK‐NE. Since NE only considers self‐diffusion, the slower dynamics in the more viscous high‐concentration systems strongly counteract the sampling effect. Additionally, already at low concentrations, the VACF is most likely already close to converged, so that the remaining uncertainty may be dominated by noise.

Similar to the ionic liquid, the NE conductivities show substantially larger values than the respective collective conductivities, emphasizing that ion–ion correlation plays an integral role even at concentrations of 1.0 M. For the highest concentration investigated, it is evident that the EH‐NE conductivity lies closer to the reference value than the corresponding EH value. This is likely again related to the error compensation between model, force field, and reference data. Additionally, the diffusion‐based conductivities overall show smaller uncertainties than the collective values, as also seen for the ionic liquid.

Altogether, it can be concluded that, for both systems, correlated movement of ions is of utmost importance for accurate prediction of charge transport. The two different approaches for the evaluation of conductivity, namely EH and GK, provide complementary insights, while EH shows higher statistical quality given the kind of simulation data accessible here.

In order to quantify the difference between collective transport and the NE approximation for the ionic conductivity, the inverse Haven ratio is evaluated. The resulting ionicities for all simulations of both electrolytes are shown in Figure S1. Overall, the ionicities are consistently well below unity, indicating that a significant fraction of the single‐particle mobility does not contribute to the macroscopic charge transport. The ${\mathrm{IL}}_{\mathrm{pol}}^{1000}$ simulation exhibits substantially smaller ionicities than the corresponding nonpolarizable simulation, demonstrating that the increased single‐particle mobility of the polarizable model does not directly translate into effective charge transport. Furthermore, ionicities show significant standard errors due to their dependence on the statistically demanding cross‐correlation contributions, while those obtained from the GK approach show larger errors than those obtained from the EH approach. A detailed discussion is provided in Section S7.2 of the Supporting Information.

#### 3.1.3 Ideal Transport Numbers

The ideal transport number describes the relative contribution of an ion to the total charge transported via self‐diffusion. These transport numbers allow for an initial straightforward interpretation of how conductivity is formally attributed to the participating ions. For the salt‐in‐solvent system, this picture will be further refined in the next sections with the inclusion of correlation effects.

**FIGURE 3:**
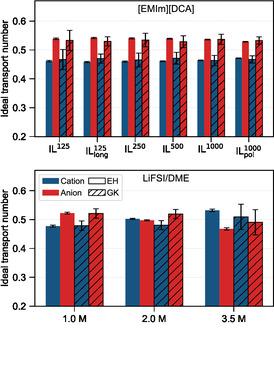
Ideal transport numbers of the cation and anion for all simulations of (top) [EMIm][DCA] and (bottom) LiFSI/DME obtained by fitting the linear regimes of the ions’ collective MSDs (EH, plain bars) or from the integral of the autocorrelation functions of the ions’ microscopic currents (GK, hatched bars).

The top and bottom panels of Figure 3 present the ideal transport numbers of the ionic liquid and the salt‐in‐solvent system, respectively.

Across all different simulations of the ionic liquid system, only minor differences in the ideal transport numbers are observed. Independent of the method (EH or GK), the anion slightly dominates ionic conductivity with ideal transport numbers between 0.52 and 0.54. This behavior was already observed in the self‐diffusion coefficients where the [DCA]^−^ ion was shown to be marginally more mobile than the [EMIm]^+^ ion (see Table 1).

It is noteworthy that the ideal transport numbers are significantly less sensitive to varying system size and force field than the absolute diffusion coefficients or conductivities. Although the absolute ion mobilities change considerably within the CL&P series as well as between CL&P and CL&Pol, the mobility ratio between cation and anion seems to be conserved.

Uncertainties stay small across all simulations as well. Especially for the transport numbers obtained from the EH methodology, the standard errors are almost negligible. Even though the GK‐based transport numbers show larger error bars, they quantitatively show the same behavior. Additionally, the uncertainties again decrease further with increasing system size (see Figure 3). This observation is consistent with the fact that the relative uncertainties for the diffusion coefficients also decrease with system size (see Table 1). These trends are then transferred to the ideal transport numbers via Gaussian error propagation.

The salt‐in‐solvent system again shows a strong concentration dependence. At low concentrations (1.0 M), the anion's contribution still exceeds the cation's contribution. However, with increasing concentration, the cationic contribution gains in relevance so much that at concentrations of 3.5 M, the cation's ideal transport number surpasses that of the anion. This development directly mirrors the trend observed for the self‐diffusion coefficients.

Here again, the MSD‐based and the correlation function–based transport numbers show the same trends. The MSD‐based values exhibit smaller standard errors and therefore cleaner resolution of the observed concentration trends. As for the ionic liquid, the uncertainties of the transport numbers reflect the behavior of the relative errors of the respective diffusion coefficients, since those carry over to the transport numbers. Again, the standard errors of the values obtained with the GK approach are significantly larger, especially for the high‐concentration system. Still, the overall qualitative trends remain the same as for the EH approach.

All in all, the ideal transport numbers for both systems appear to be more stable than the absolute diffusion coefficients or NE conductivities. This is expected, since systematic influences on the self‐contributions cancel out during normalization. Changes in mobility that affect both ions are therefore less visible in the ideal transport numbers. Due to this observed stability and easy accessibility, they are helpful for assessing the relative distribution of charge transport independently of the correlation effects discussed later.

At the same time, it is necessary to emphasize that, since ideal transport numbers are solely based on self‐diffusion, they cannot display to which extent cross‐correlations between ions change the contributions of each species to the macroscopic charge transport. Thus, they reflect a helpful middle‐ground between self‐diffusion and the collective conductivity but do not describe real charge transport in its full complexity.

### 3.2 Salt‐in‐Solvent System—Reference Frame Effects and GK Integrals

**FIGURE 4:**
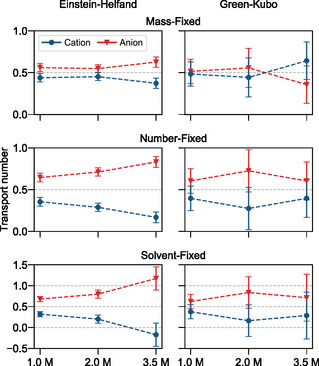
Real transport numbers calculated using the Einstein–Helfand (left) and Green–Kubo (right) methods with different reference frames for the LiFSI/DME systems.

As recently shown by Zhang and coworkers [70] for polymer electrolytes, the transport numbers are strongly dependent on the reference frame used in the calculation of the ionic conductivity. Furthermore, as discussed in previous literature, in pure ionic liquids and salt melts, the transport numbers collapse to purely analytic fractions of ion properties such as mass or volume, depending on the arbitrary choice of the reference frame [36, 99, 100]. In these systems, the transport numbers therefore carry no additional physicochemical meaning. Therefore, in Figure 4, we only show the transport numbers for Li^+^ and FSI^−^ in the salt‐in‐solvent system obtained using different reference frames with the EH and GK approaches. For completeness, “real” transport numbers in the barycentric reference frame for [EMIm][DCA] are listed in Table S12 in the Supporting Information.

First, as in the ionic conductivity calculations shown above, the transport numbers obtained using the GK approach present large error bars, making it difficult to see any clear trend for the salt concentration effect in transport numbers or even to understand the reference frame impact, Figure 4. On the other hand, the smaller error bars in the EH calculations show that the increase in the salt concentration leads to the decrease in the Li^+^ transport numbers in all reference frames.

Although the trends are similar across all reference frames in the EH calculation, the values of the Li^+^ transport numbers differ, with the mass‐fixed frame yielding the largest and the solvent fixed frame the smallest values. Zhang and coworkers [70] reported similar behavior, in which they observed a decrease in *t*_Li_^+^ when the solvent‐fixed reference frame was used, which is due to the smaller fraction of solvent molecules at higher salt concentrations and consequently an “amplification” of anion flow effect in the Li^+^.

Unlike Zhang et al. [70], the change in the reference frame in Figure 4 did not alter the qualitative trends with respect to salt concentration; i.e., the increase in salt concentration leads to the decrease in the cation transport number. This demonstrates that the influence of the reference frame on transport numbers should be evaluated for every material class independently. It should be noted that only the solvent‐fixed reference frame yields negative values for the Li^+^ transport number. Although this effect may appear unexpected, similar results have been observed in other electrolyte systems [101, 102] and rationalized in terms of the strongly coupled motion of the cation–anion species. Therefore, transport numbers must be interpreted with caution.

**FIGURE 5:**
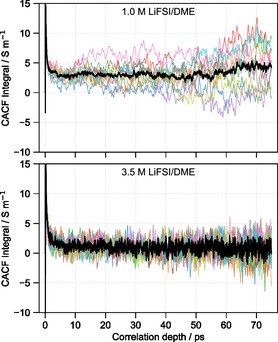
Integral of the autocorrelation functions used to calculate the ionic conductivity within the GK approach for the LiFSI/DME system at 1.0 and 3.5 M. The black curves are the averaged integrals, while the colored ones are moving averages of the individual replicas over a 200‐fs window.

The large error bars present in the GK approach shown above are due to the increased sampling requirement and the difficulty in identifying the plateau in the integral of the autocorrelation function, which may also be due to insufficient sampling. As shown in Figure 5, even when using 10 independent replicas, the integrals do not necessarily yield a smooth and linear behavior. However, when comparing the 1.0 and 3.5 M systems, a more consistent behavior between independent replicas is observed at higher concentration, resulting in smaller bootstrap uncertainties. This can be rationalized by the increased number of ions present in the high‐concentration system, allowing for more representative ion dynamics through enhanced self‐averaging, as a larger region of phase space is effectively sampled within a single trajectory. Nevertheless, the 3.5 M system shows stronger fluctuations within the CACF integrals of individual trajectories, most likely due to the higher viscosity and thereby slower relaxation times. These lead to longer lived correlations in the charge current and consequently to a reduced effective number of statistically independent time origins for the correlation function, thus amplifying the variance of the long‐time tail around zero. Furthermore, the differences in CACF integrals between the systems in Figure 5 show that the choice of simulation length, correlation depths, and number of replicas must take into account the electrolyte properties, such as viscosity and mass transport, as well as the size of the simulated system.

### 3.3 Salt‐in‐Solvent System—Fit Method

**FIGURE 6:**
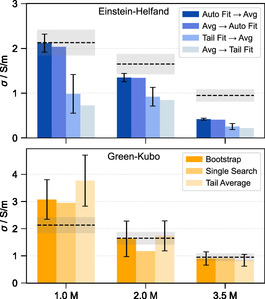
Conductivities for the LiFSI/DME system obtained from the Einstein–Helfand (top) or Green–Kubo approach (bottom) with different fitting strategies. Experimental reference values are shown as dashed lines and were interpolated from refs. [86, 87]; see Section S4.

It has been observed [45, 94] that multiple independent replica simulations are required to obtain reliable conductivities from either the EH or GK approach. However, there exist several approaches for combining the insights gained from those independent replicas into a single representative estimate. In practice, one might calculate individual estimates for each replica, respectively, and average the obtained values or, alternatively, first average the CMSD or CACF and then extract a single estimate from the averaged function. Formally, both approaches should yield the same result. In practice, however, the optimal diffusive time window of the CMSD or the plateau region in the CACF's integral may differ between replicas. When uncertainty‐aware algorithms are used to identify appropriate time windows, the final result is no longer independent of the order of operations. Figure 6 compares the conductivity estimates obtained from different strategies within the EH and GK approach, respectively. Most workflows reproduce the same qualitative trend of decreasing conductivity with increasing salt concentration. However, the quantitative differences between the estimates can be significant.

The top panel of Figure 6 compares four EH conductivity estimates obtained by combining either automated fit‐window identification or a fixed‐tail fit, treating the second half of the CMSD as fit window (see Section S7.5), with either replica‐first fitting or averaging prior to fitting. It is apparent that within the EH approach the fitting strategy is more important than the order of averaging. For both orders of fitting and averaging, the estimates obtained using fit‐window identification are nearly identical for all three salt concentrations and lie within the range of the uncertainty. In contrast, the simpler fixed‐tail fit approach leads to significantly lower conductivity estimates. The relative decrease at 1.0 M is over 50% compared to the earlier estimates and, while the absolute gap decreases, it remains large also at higher concentrations. Furthermore, the fixed‐tail fit approach results in a higher uncertainty, which implies a stronger scatter between replicas in the tail region compared to the automatically detected diffusive regions. A fixed fit window may still yield acceptable estimates if the chosen window appropriately captures the diffusive regime across all replicas. However, the optimal window location is system dependent, and intermediate‐time deviations from linearity can bias the fitted slope and distort the final estimate. The results in Figure 6 demonstrate the strength of the automated window detection, as the algorithm can effectively avoid such distortions and yield robust estimates across systems.

The bottom panel of Figure 6 compares three GK conductivity estimates. In all cases, the conductivity is obtained as the mean value of the integrated CACF over a selected plateau window. The first estimate uses bootstrap resampling with automated plateau detection for each sample, the second is obtained from a single plateau detection on the integral of the averaged CACF, and the third estimate uses a fixed‐tail window from 50 to 75 ps applied to the individual replicas (see Section S7.3). For the fixed‐tail estimate, the integral values were first averaged over the 50–75 ps interval for each individual replica, and the final conductivity and standard error were then obtained as the unweighted mean and standard error over the resulting tail averages. Note that the automated plateau detection on a single replica's CACF is unlikely to return a meaningful time window due to the significant noise in the long‐time region. Averaging the CACF first reduces noise, but the cross‐replica standard error is lost. All approaches yield qualitatively similar results that lie within each other's uncertainty range. The bootstrap approach gives estimates best aligned with the reference values.

Overall, the results show no significant differences between average‐first and replica‐first strategies. The main advantage of the latter is therefore not the accuracy of the conductivity estimate itself, but the uncertainty estimate. Processing replicas individually or using bootstrap sampling yields a meaningful standard error, whereas processing the averaged CMSD or CACF does not.

### 3.4 Ionic Liquid System—Sampling Dependence and Size Effects

#### 3.4.1 Phase Space Sampling

In order to better assess the reliability of the calculated ionic conductivities in the ionic liquid system, we investigated the influence of two approaches for increasing sampling within MD simulations. For this purpose, the 10 independent 100 ns replicas for a given simulation condition (i.e., IL^1000^) were split again into 10 smaller trajectories of 10 ns. Each of these small trajectories was then used to estimate the ionic conductivity with the use of the robust EH method explained in the previous section, with a maximum correlation depth of 3 ns, i.e., 30% of the simulation time length.

Based on those 10 × 10 segments, two averaging strategies were compared. First, the weighted means of the obtained conductivities from the 10 partial trajectories of each replica were calculated and their scaled standard error was estimated. This yields one conductivity value with an associated standard error per replica. In the second approach, the segments from the different replicas were grouped based on their segment index. Then, the weighted mean was calculated for each group, so that every index relates to exactly one value together with a standard error, and each index contains data from every single replica. The first approach primarily captures fluctuations within a single long trajectory, whereas the second method deals with variations between independent replicas.

**FIGURE 7:**
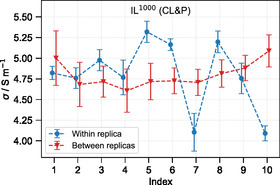
Exemplary display of varying weighted means obtained from the averaging of segments within replicas (blue) vs. averaging segments across replicas (red) for the IL^1000^ system. The index on the *x*‐axis denotes the replica or segment index, respectively.

In Figure 7, the comparison of the two approaches is shown for the set of IL^1000^ simulations. It can easily be seen that both approaches yield different amounts of scatter along the index axis. The averages over the segment groups (containing contribution from all replicas) show relatively small variations and lie within a close range of 4.6–5.1 S m^−1^. In contrast, the values averaged over all segments within a replica (blue curve) fluctuate significantly more, displaying values in a range of about 4.1–5.3 S m^−1^. Additionally, it is evident that the error bars for the values obtained from across‐replica averaging are larger than for the averages within replicas. Therefore, it can be concluded that the different segments of a trajectory yield relatively consistent values, whereas the spread between replicas is substantial. This suggests that uncertainties connected to dynamic quantities like conductivity are not primarily dominated by short‐term fluctuations within a trajectory but more significantly by the accessible regions of phase space.

These findings are reinforced by the systematic evaluation of standard errors in Table 3. For both approaches, the averages of the standard errors (over all indices) are shown for both across‐ and within‐replica averaging. Without exception, the mean errors for the averaging across replicas are larger than those obtained when averaging segments within replicas. The fact that all system sizes and both force fields exhibit this trend shows that starting multiple replicas from different initial conditions introduces greater variability in the ion dynamics than observed within single long trajectories [45].

**TABLE 3:** Comparison of mean standard errors from segment‐wise (SE_across rep._) and replica‐wise (SE_within rep._) averaging for all [EMIm][DCA] simulations. All values are given in S m^−1^.

Simulation	SE_across rep._	SE_within rep._
IL^125^	0.2522	0.1675
${\mathrm{IL}}_{\mathrm{long}}^{125}$	0.2353	0.1751
IL^250^	0.2330	0.1749
IL^500^	0.2116	0.1521
IL^1000^	0.2093	0.1377
${\mathrm{IL}}_{\mathrm{pol}}^{1000}$	0.2046	0.1518

Although a single long trajectory is able to capture many configurations, it is still constrained by the kinetics that limit finite simulations to a small part of the total phase space [45]. Several independent replicas with different starting configurations can, on the other hand, allow for wide sampling of all possible microscopic states and can therefore yield a representative set of configurations drawn from the whole phase space. The latter can help to obtain more reliable estimates for the variability of a macroscopic system. Smaller uncertainties within single replicas should therefore not be understood as more precise estimations but rather as an expression of the partially underestimated uncertainties and limited phase space coverage.

For the estimation of conductivities, this means that independent replicas are not only necessary for an increase in sampling but especially for capturing replica‐to‐replica variability. In particular for collective transport properties that are sensitive to rare and long‐range correlated movement patterns, broad sampling of the phase space is of higher importance than refining local patterns within a single trajectory.

#### 3.4.2 Correlation Depth Influence

**FIGURE 8:**
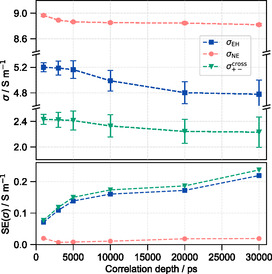
EH conductivity, NE conductivity, and anion–cation cross‐contribution as obtained from the ${\mathrm{IL}}_{\mathrm{pol}}^{1000}$ simulation with different maximum correlation depths (top) and the corresponding standard error (bottom).

To assess the EH conductivity's dependence on the maximum correlation depth considered when building the CMSD, we varied that parameter from 1 to 30 ns (and up to 240 ns for the ${\mathrm{IL}}_{\mathrm{long}}^{125}$ simulation) for all investigated systems. Figure 8 shows the EH and NE conductivities, as well as the anion–cation cross‐contribution calculated from the ${\mathrm{IL}}_{\mathrm{pol}}^{1000}$ simulation with different maximum correlation depths. For the other systems, see Section S7.8.

A general observation across all systems is that the NE conductivity shows little variance with the correlation depth. Both its value and the corresponding standard error remain remarkably consistent with increasing time depth. The standard error itself is comparatively small and an order of magnitude lower than that of the EH conductivity and anion–cation cross‐contribution. Similar behavior is observed for the cation's and anion's self‐contributions. This shows that the uncorrelated single‐particle contributions converge quickly at relatively low correlation depths and are not the main source of the EH conductivity's uncertainty.

In contrast, the EH conductivity is observed to vary more strongly with the maximum correlation depth than the NE conductivity. This is consistent across all investigated systems. At the same time, its standard error grows steadily with increasing correlation depth. This may be attributed mainly to the distinct cross‐terms and the anion–cation cross‐contribution, while the cross‐terms between like‐charged ions are somewhat less sensitive. The relative increase in the EH conductivity's standard error is significantly stronger than the change in its mean value. Figure 8 therefore shows that increasing the maximum correlation depth beyond a certain fraction of the trajectory length tends to increase statistical noise more than it improves the conductivity estimate.

Taking into account all system sizes together (see Figure S18), a common pattern emerges. At small correlation depths, the total EH conductivity tends to be slightly higher and then decreases as the short‐time contribution in the CMSD to the final estimate is reduced. However, at larger depths, no clear monotone convergence is observed anymore. Instead, the conductivity estimates fluctuate and often grow again as their SE increases.

The comparison between the simulations of IL^125^ and ${\mathrm{IL}}_{\mathrm{long}}^{125}$ supports this interpretation. While the shorter IL^125^ simulation shows a stronger irregular depth dependency, the longer ${\mathrm{IL}}_{\mathrm{long}}^{125}$ simulation shows that the EH conductivity remains nearly constant over a wide correlation depth range while its standard error increases. The fluctuations at larger correlation depths therefore do not point to a continued systematic drift of the conductivity but rather to an increasingly noise‐dominated regime.

Overall, the data demonstrate that the convergence of the EH conductivity is controlled much more by the long‐time behavior of the distinct ion cross‐correlation terms than by the self‐contributions. For the investigated systems, moderate correlation depths are sufficient to obtain stable central values, while larger depths mainly increase uncertainty.

#### 3.4.3 Size Effects

**FIGURE 9:**
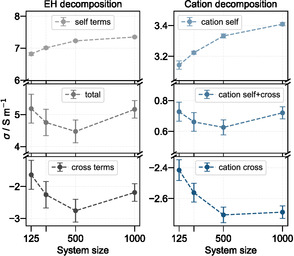
Decomposition into self‐ and cross‐terms of the (left) total conductivity and (right) the cation contributions in the barycentric frame of reference, as obtained from the CL&P simulations of [EMIm][DCA] containing different amounts of ion pairs.

Since diffusion coefficients and therefore also the self‐contributions of the ions to the conductivity have already been shown to be system size dependent, the question arises of how the other parts that make up the total conductivity behave with varying system size. For the purpose of investigating this, the left panels in Figure 9 show the sum of all self‐terms, the total conductivity, and the sum of all cross‐terms obtained for the four sets of CL&P simulations for the ionic liquid systems that differ in the number of simulated ion pairs. First of all, the expected size dependence of the self‐terms is shown again with differences between the values that far exceed the related uncertainties. In contrast to this, the total conductivity exhibits strongly overlapping standard errors, making it difficult to reliably deduce any trends. The combination of sensitive self‐terms and insensitive total conductivities suggests that cross‐terms (at least partially) compensate the size dependence of the self‐terms. Regarding the data in Figure 9, the sum of the cross‐terms also shows large uncertainties, making it difficult to support this hypothesis confidently. However, since at least for small system sizes there seems to be a slight tendency for the cross‐terms to decrease with system size, we took a closer look at the isolated cation contributions shown in the right panels of Figure 9.

While the self‐term shows the same trend as seen before, the cross‐term of the cation carries smaller standard errors, and thus, its size dependence can be evaluated with more confidence. Note that the considered separated cation–cation cross‐term is (in contrast to the sum of all cross‐terms shown before) strongly reference frame dependent. However, since here we are mainly interested in its dependence on the size of the simulation box, the absolute values are not of great relevance. Within the context of the uncertainties, the cross‐term shows a clear system size dependence, by decreasing from around −2.4 to −2.7 S m^−1^ with a maximum standard error of only ±0.07 S m^−1^. Thus, the total change of the cross‐term, going from 125 ion pairs to 1000 ion pairs, lies in the same range as the change in the self‐term. The total conductivity varies only within a small range of 0.62–0.73 S m^−1^, showing no prominent trend, but still overlapping error bars. All in all, this can be interpreted as at least a partial compensation effect between self‐ and cross‐terms regarding finite size effects.

If the goal is to obtain definitive evidence regarding the size effects on the total conductivity, as well as all of its components, even more extensive sampling in the form of additional replicas and/or even longer simulations is required. Note that these findings can differ depending on the investigated systems. Therefore, observed trends should not be interpreted as general trends until more extensive investigations across different systems are carried out.

## 4 Conclusion

In this work, we presented the conduct module now implemented in TRAVIS and validated its functionality with the example of two different electrolyte systems, the ionic liquid [EMIm][DCA] and the ether‐based electrolyte LiFSI/DME. The module allows for the calculation of ionic conductivities as well as derived quantities like ideal transport numbers and ionicities, using both the EH and GK approaches.

Our calculations agree well with experimental reference data from the literature and show a qualitative agreement between EH and GK, demonstrating a robust basis for the investigation of collective charge transport. The EH approach showed better numerical stability with smaller uncertainties within our simulation setup, which can be attributed to limited sampling in the corresponding short trajectories used for the GK calculations, resulting in noisy CACF integrals. Therefore, the EH approach is preferred for simulations that do not fulfill the GK approach's requirements for extensive sampling, but nonetheless, the GK method can be used as a valuable complementary approach for methodological consistency checks and qualitative trend analysis.

The comparison of self and collective transport shows for both systems that ion–ion correlation effects play a crucial role in the overall charge transport. The NE approximation systematically overestimates the true ionic conductivity, as it is solely based on self‐movement and neglects collective ion motion. Ionicities reflect this behavior, significantly falling below unity. When comparing the two different force fields, CL&P and CL&Pol, we find that the predicted self‐diffusion coefficients are higher in the polarizable model, whereas the collective diffusion coefficients do not show a significant difference. Instead, the ionicities are lower for the CL&Pol model, indicating a stronger correlation between ions in this model. This proves that single‐particle mobilities are not necessarily translated into collective transport properties. Therefore, the investigation of correlation effects is of great importance.

Ideal transport numbers prove to be mostly insensitive to parameters like system size, force field, or even the approach used to obtain the data from the simulations. However, since they lack correlation information, they may not fully capture the complexities of the charge transport phenomena. Therefore, for the LiFSI/DME system, we also calculated “real” transport numbers. These properties are strongly reference frame dependent, which can lead to different interpretations of the ionic transport behavior, particularly between experiment and simulations. For this purpose, the new conduct module in travis provides the option to calculate transport numbers in different reference frames, which can help to bridge the gap between simulations and experiments. We found that in the solvent‐fixed reference frame, the transport numbers for Li^+^ can even become negative, highlighting the importance of the chosen reference frame in interpreting ionic transport behavior.

Additionally, we focused on the methodological aspects of obtaining robust conductivity estimates and uncertainties from a set of independent trajectories. In agreement with literature, it was shown that not only the length of a simulation but also the number of independent replicas is of high importance when aiming for reliable conductivity values, and these variables should be defined independently for every simulated material. Splitting long trajectories into many short ones shows a systematic underestimation of the true uncertainty, mainly arising from the limited sampling of the phase space. Independent replicas, on the other hand, capture the true replica‐to‐replica variability and thus represent the relevant uncertainty of collective transport properties much more realistically. For the reliable determination of conductivities, therefore, several independent replicas are not only advantageous but methodically essential.

Within the EH formalism, we demonstrated that the automatic time window identification of a suitable diffusive regime significantly improves the robustness of the results. Accordingly, for the GK approach, bootstrap‐based plateau identification represents the statistically most sensible procedure. Overall, these results show that concrete numerical evaluation strategies can play a crucial role in the reliability of the obtained transport properties and their corresponding uncertainties.

The analysis of correlation depth and system size effects provides important insights for future applications. While moderate maximum correlation depths (10 ns with good sampling, 10% of the total simulation length) are sufficient to obtain reliable EH conductivities for the investigated systems, larger correlation depths mainly lead to increased statistical noise without significantly improving mean values. In the case of the ionic liquid cation, we additionally found that the strong system size dependence of the self‐contribution to the conductivity is partially compensated by the corresponding cross‐term. Therefore, possible finite size effects in the total conductivity are much harder to resolve than in the self‐diffusion coefficients. Since sufficient correlation depths and potential system size dependencies can differ from system to system, these should be tested for any investigated system separately. In order to obtain a reliable interpretation of the size effects in complex collective transport properties, extensive statistical sampling is essential.

In addition, since both methods, EH and GK, provide similar results and conclusions, the choice between them should be made taking into account their own requirements and the available resources. For example, to obtain reliable values, EH requires longer simulation times to achieve a true diffusive regime, which leads to longer computation times. On the other hand, GK requires shorter simulation times, but the velocities must be saved more frequently, which leads to a larger amount of stored data. Therefore, the user should carefully decide which kind of analysis is the most suitable.

In conclusion, the conduct module available in TRAVIS is an efficient and methodically versatile tool to calculate ionic conductivities for different systems, implementing both the EH and GK approaches and allowing the user to choose between different reference frames. Aside from that, we were able to show which methodological aspects are crucial for obtaining reliable and physically meaningful results. Among those are the use of independent replicas, an adaptive window or plateau selection, a careful interpretation of reference frame dependent transport quantities, and an explicit consideration of collective ion–ion correlations. Overall, this work not only provides a practical tool for analyzing ionic systems, but also a systematic methodological framework for their reliable evaluation.

## Funding

This study was supported by Deutsche Forschungsgemeinschaft (CRC 1639 NuMeriQS, project no. 511713970, BR 5494/3‐1), FWO and F.R.S.‐FNRS (EOS 40007515), Fundação de Amparo à Pesquisa do Estado de São Paulo (2024/19559‐2, 2026/01706‐4, 2022/05652‐5, 2017/11631‐2, 2018/21401‐7), Breakthrough Electrolytes for Energy Storage Systems (DE‐SC0019409), and Agência Nacional do Petróleo, Gás Natural e Biocombustíveis.

## Conflicts of Interest

The authors declare no conflicts of interest.

## Code Availability Statement

The conductivity analysis presented in this work is implemented in the open‐source post‐processing package TRAVIS, available at http://www.travis‐analyzer.de/. Additional Python scripts used for data processing are openly available at https://github.com/kirchners‐manta/conductivity_tools.

## Supporting information

Supplementary Material

## Data Availability

The data that support the findings of this study are available from the corresponding authors upon reasonable request.
