# Supplementary material for: Lessons Learned on Obtaining Reliable Conductivity Estimates From Molecular Dynamics Simulations
Source: Chemphyschem. 2026 Jul 24;27(14):e70477. doi: 10.1002/cphc.70477 (PMC13400156; doi:10.1002/cphc.70477)
Supplement: Supplementary file 1 — Supplementary Material [file CPHC-27-e70477-s001.pdf]

Supporting Information: Lessons learned on obtaining reliable conductivity estimates from molecular dynamics simulations

Paul Zaby,[1] Johannes Ingenmey*,[1] Tuanan C. Lourenço*,[2] Yong Zhang,[3] Juarez L. F. Da Silva,[4] Martin Brehm,[5] Edward J. Maginn,[3] Barbara Kirchner*[1]

[1] Mulliken Center for Theoretical Chemistry, University of Bonn, Germany.

[2] Department of Physics and Mathematics, Institute of Chemistry, São Paulo State University, Brazil.

[3] Department of Chemical and Biomolecular Engineering, University of Notre Dame, US.

[4] São Carlos Institute of Chemistry, University of São Paulo, Brazil.

[5] Department of Chemistry, Paderborn University, Germany.

*E-mail: ingenmey@uni-bonn.de; tuananclourenco@gmail.com; bkirch@uni-bonn.de;

Contents

S1 Introduction **2**

S2 List of Variables and Abbreviations **2**

S3 Theoretical Approach and Computational Details **4**

S3.1 Force Field Models 4

S3.2 Molecular Dynamics Simulation Details 4

S4 Reference Conductivities for LiFSI/DME **6**

S5 Diffusion Coefficients **7**

S6 Single Trajectory Green-Kubo Calculations in TRAVIS **10**

S7 Additional Results **11**

S7.1 Total Conductivities 11

S7.2 Inverse Haven-Ratio - Ionicities 11

S7.3 Charge Current Autocorrelation Functions 15

S7.4 Diffusion coefficients 19

S7.5 Collective MSD 20

S7.6 Transport Numbers 23

S7.7 Replica vs Segments 25

S7.8 Correlation Depth 26

S7.9 Decomposition 27

S7.10 Discrepancy between EH and GK results - Additional Analyses 31

S1 Introduction

In this supporting information, we present the list of variables and abbreviations used in the main manuscript, the theoretical approach and computational details for the classical molecular dynamics simulations, the references conductivities used in the validation of the salt-in-solvent systems, the theoretical description of the Einstein and Green–Kubo formulation for the self-diffusion coefficients, as well as additional/complementary results for the main manuscript.

S2 List of Variables and Abbreviations

Abbreviation	Meaning
MD	molecular dynamics
[EMIm][DCA]	1-ethyl-3-methylimidazolium dicyanamide
LiFSI	lithium bis(fluorosulfonyl)imide
DME	1,2-dimethoxyethane
EH	Einstein–Helfand method
GK	Green–Kubo method
NE	Nernst–Einstein approximation
MSD	mean square displacement
VACF	velocity autocorrelation function
CACF	charge current autocorrelation function
CMSD	collective mean square displacement
FFT	fast Fourier transform
SE	standard error

Variable	Meaning
e	charge of an electron
V	simulation box volume
k_B	Boltzmann constant
T	temperature
C	number of charged species in the system
z_k	integer charge of ionic species k in units of e
N_k	number of ions of type k
N_+	number of positively charged ions
N	total number of ions
D_k	self-diffusion coefficient of species k
t_max	total number of timesteps
$\mathrm{MSD}^\mathrm{col}(\tau)$	collective MSD at correlation depth τ
$\vec{r}_i(t)$	position vector of ion i at time t
$\Delta\vec{r}_i(t,\tau)$	displacement vector of ion i from time t to time $t+\tau$
$\lVert\cdot\rVert_2$	euclidean norm of a vector
$\vec{J}(t)$	system charge current at time t
$\vec{J}_k(t)$	charge current of species k at time t
$\vec{J}_{kl}(t)$	charge current of the subsystem made up from ions of species k and l
$\vec{v}_i(t)$	velocity of ion i at time t
σ_NE	Nernst–Einstein conductivity

$\sigma_{\mathrm{tot}}^{\mathrm{EH}}$	Einstein–Helfand total conductivity
$\sigma_{\mathrm{tot}}^{\mathrm{GK}}$	Green–Kubo total conductivity
σ_k^{self}	self-contribution of species k to the ionic conductivity
$\sigma_k^{\mathrm{self,GK}}$	GK self-contribution of species k to the ionic conductivity
σ_k^{EH}	collective EH contribution from all ions of type k (self and cross)
σ_k^{GK}	collective GK contribution from all ions of type k (self and cross)
σ_+^{EH}	collective EH contribution from all cations (self and cross)
$\sigma_{kl}^{\mathrm{EH}}$	total EH contribution from all ions of type k and l and their cross contribution
$\sigma_{kl}^{\mathrm{GK}}$	total GK contribution from all ions of type k and l and their cross contribution
$\sigma_{kl}^{\mathrm{cross}}$	contribution of cross correlations between ions of type k and ions of type l
σ_+^{self}	self-contribution of all cations
σ_-^{self}	self-contribution of all anions
$\sigma_{++}^{\mathrm{cross}}$	contribution of the cross correlations between all cations
$\sigma_{--}^{\mathrm{cross}}$	contribution of the cross correlations between all anions
$\sigma_{+-}^{\mathrm{cross}}$	contribution of the cross correlations between all cations and all anions
σ_r	conductivity obtained from replica r
t_i^{id}	ideal transport number of species i
t_i^{real}	"real" transport number of species i
H^{-1}	inverse Haven ratio (ionicity)
$\vec{v}_i^{(\alpha)}(t)$	velocity of ion i at time t in reference frame α
$\vec{v}_i^{\mathrm{COM}}(t)$	center of mass velocity of particle i at time t
$\vec{w}^{(\alpha)}(t)$	reference velocity at time t in frame α
m_i	atomic mass of atom i
M	total mass of the system
N_{tot}	total number of particles (molecules and ions) in the system
N_{solv}	total number of solvent molecules in the system
$\Delta\vec{r}_i^{(\alpha)}(t,\tau)$	displacement vector of ion i from time t to time $t+\tau$ in reference frame α
$\Delta\vec{R}^{(\alpha)}(t,\tau)$	displacement vector of the origin of reference frame α from time t to time $t+\tau$
$\vec{J}^{(\alpha)}(t)$	system charge current at time t in reference frame α
$\mathrm{MSD}_k^{(\alpha)}(\tau)$	mean square displacement of species k at correlation depth τ in reference frame α
$\mathrm{MSD}_r^{\mathrm{col}}(\tau)$	collective MSD at correlation depth τ obtained from replica r
N_r	number of replicas
$\Delta\mathrm{MSD}_r^{\mathrm{col}}(\tau)$	standard error of the collective MSD at depth τ from replica r
$\Delta\sigma_r$	fit-error ob the conductivity obtained from replica r
σ_{eff}	effective conductivity obtained from weighted average over all replica conductivities
$\Delta\sigma_{\mathrm{eff}}$	standard error of effective conductivity
w_r	weight of conductivity obtained from replica r
χ^2_{red}	reduced Chi-squared statistic

$\Delta\sigma_{\mathrm{eff,scaled}}$	standard error of effective conductivity scaled using the Chi-squared statistic
$\mathrm{MSD}_{\mathrm{eff}}^{\mathrm{col}}(\tau)$	effective CMSD at correlation depth τ from weighted average over replica CMSDs

S3 Theoretical Approach and Computational Details

To validate and illustrate the capabilities of the `conduct` module implemented in TRAVIS, as well as the approaches proposed in this manuscript, we selected two different systems: the ionic liquid 1-ethyl-3-methylimidazolium dicyanamide, [EMIm][DCA], and the salt-in-solvent electrolyte lithium bis(fluorosulfonyl)imide in monoglyme, LiFSI/DME, in three different salt concentrations (1.0 M, 2.0 M and 3.5 M). In the following, we provide all the technical details about the force field models and the MD simulation protocol.

S3.1 Force Field Models

Choosing the correct force field is one of the key points in molecular dynamics simulations, especially when the focus is on calculating transport properties. Then, to ensure a good description of the ionic conductivity in the simulations, in both LiFSI/DME and [EMIm][DCA] simulations, we employed the polarizable force field CL&Pol developed by Canongia and Pádua groups [1,2]. The CL&Pol is the polarizable version of the CL&P which is based on the OPLS force field and has been widely used in MD simulations of electrolyte and ionic liquid systems [3–6] with reasonable accuracy for transport properties. In the CL&Pol force field, Drude particles are attached to the non-hydrogen atoms to model the atomic charge polarizabilities. A more detailed description of this force field can be found in the literature. [1,2]

In addition, to understand the impact of the force field in the ionic conductivity calculations, we also performed MD simulations for [EMIm][DCA] using the CL&P model, the non-polarizable version of the CL&Pol, with the atomic charges scaled by a factor of 0.8. The use of scaled charges in non-polarizable MD simulations is a straightforward approach used in the simulation of electrolytes and ionic liquids to account for polarizability and charge transfer effects. The relaxed structures for all chemical species in the simulations, as well as all the force field parameters, were obtained directly from the CL&Pol database using the fftool and polarizer scripts from the Pádua group. [1,2,7]

S3.2 Molecular Dynamics Simulation Details

For all systems, the initial MD simulation boxes were built using the fftool script and PACKMOL software. [8] To obtain a deeper understanding of the effects of system size on ionic conductivity calculation, we considered four different system sizes for the [EMIm][DCA] ionic liquid, namely 125, 250, 500 and 1000 ion pairs. For LiFSI/DME we only considered one system size with three different concentrations, where we fixed the total number of particles (LiFSI + DME) at 1500 in all simulations. The box compositions are shown in Table S2.

Table S2. MD simulation box compositions for all simulated systems.

Simulation	$[EMIm]^+$	$[DCA]^-$	Run 1 / ns	Run 2 / ns
IL^{125}	125	125	100	0.1
IL^{125}_{long}	125	125	800	0.2
IL^{250}	250	250	100	0.1
IL^{500}	500	500	100	0.1
IL^{1000}	1000	1000	100	0.1
IL^{1000}_{pol}	1000	1000	100	0.1

Simulation	Li^+	$[FSI]^-$	DME	Run 1 / ns	Run 2 / ns
1.0 M	150	150	1350	100	0.1
2.0 M	300	300	1200	100	0.1
3.5 M	500	500	1000	100	0.1

First, for all systems listed in Table S2, five independent simulation boxes with random configurations were generated using PACKMOL. For the ionic liquid system, the initial configurations were then (i) relaxed using the conjugate gradient algorithm, followed by (ii) a 1.0 ns NVT equilibration at 700 K. Subsequently, (iii) the temperature was decreased to 353.15 K in a 1.0 ns NpT simulation at 1.0 atm, then (iv) additional 10.0 ns were performed with the NpT ensemble at a fixed temperature of 353.15 K, in which the last 5.0 ns were used to obtain the average equilibrium box volume.

To ensure the statistical robustness of our data, 10 new simulation boxes were generated using PACKMOL and submitted to the MD protocol stages (i) to (iii). In the end, (v) 2.0 ns NpT simulations at 1.0 atm and 353.15 K were performed and the simulation boxes are scaled to the NpT average volume obtained in stage (iv). In this way, we ensure that all the 10 replicas simulation are totally independent and have the same density. Finally, (vi) the production runs were performed in the NVT ensemble at 353.15 K for 111 ns (Run 1), in which the first 11 ns were considered as equilibration part and discarded and the remaining trajectory used for the EH analysis. For the GK analysis, additional production runs were conducted for 0.1 ns (Run 2). In the IL^{125}_{long} simulations, production runs were extended to 811 ns, with again the first 11 ns considered as equilibration, while the additional runs required for the GK analysis where 0.2 ns long.

The salt-in-solvent systems were simulated using the same MD protocol as described above for the [EMIm][DCA], but with different temperatures. Simulation stage (ii) was performed at 500.00 K, while for all the other stages a temperature of 333.15 K was used. Table S2 highlights the production simulations used in each system.

In all the CL&Pol simulations above, the temperature and pressure were controlled by the temperature-grouped Nosé—Hoover thermostat, TGNH, using damping parameters of 0.10 ps and 1.00 ps, respectively. As recommended in the seminal works on the CL&Pol force field, for the Drude particles the temperatures were kept at 1.0 K with damping parameters of 0.025 ps. Furthermore, in

the [EMIm][DCA] CL&Pol simulations we used only the Thole damping function,[9–11] while for the LiFSI/DME simulations both Thole function and the Tang–Toennies dispersion damping function were used.[4,12] For non-polarizable simulations, the pressure and temperature were handled by the Nosé–Hoover thermostat and barostat with damping parameters of 0.10 ps and 1.00 ps, respectively.

Non-bonded short range and Coulombic interactions were handled with a 1.20 nm cutoff and the Particle-Particle Particle-Mesh, respectively. The velocity-verlet algorithm was used to integrate the equations of motion with a 1.00 fs timestep.

For all Run 1 simulations (see Table S2), with the exception of the $\mathrm{IL}_{\mathrm{long}}^{125}$ system, the atomic positions were dumped every 1000 steps. For the $\mathrm{IL}_{\mathrm{long}}^{125}$ simulations, only every 4000th step was saved. In the case of the Green–Kubo (Run 2) simulations, atomic positions, and velocities were dumped every timestep. All simulations were performed using the LAMMPS (Large-scale Atomic/Molecular Massively Parallel Simulator) package, version 23Jun2022.[13]

S4 Reference Conductivities for LiFSI/DME

While some experimental ionic conductivity data for LiFSI in DME can be found in literature (see Table S3),[14,15] reference values at the simulated conditions could not be found. Qian *et al.*[14] measured the concentration dependence at a temperature of $T = 298.15\,\mathrm{K}$, and Zhao *et al.*[15] measured the temperature dependence at a concentration of $c = 1.0\,\mathrm{mol\,L^{-1}}$. Reference values in this work were estimated from these two datasets under the approximate separability assumption

$$\sigma(c,T) \approx f(T_0 \to T)\ \sigma(c,T_0)$$

and

$$\sigma(c,T) \approx f(c_0 \to c)\ \sigma(c_0,T),$$

where $\sigma(c,T)$ is the conductivity at concentration c and temperature T and f is a scaling factor that describes the relative change in the conductivity from temperature T_0 to T or concentration c_0 to c.

The scaling factor $f(298.15\,\mathrm{K} \to 333.15\,\mathrm{K})$ was calculated by fitting the temperature-dependent dataset to a Vogel–Fulcher–Tammann type equation

$$\sigma_{\mathrm{VF}} = \sigma_0 \exp\left\{-\frac{B}{T - T_{\mathrm{VF}}}\right\}$$

and then

$$f(298.15\,\mathrm{K} \to 333.15\,\mathrm{K}) = \frac{\sigma(1.0\,\mathrm{mol\,L^{-1}}, 333.15\,\mathrm{K})}{\sigma_{\mathrm{VF}}(1.0\,\mathrm{mol\,L^{-1}}, 298.15\,\mathrm{K})} = \frac{2.33}{2.03} = 1.148$$

By performing a linear fit on the concentration-dependent dataset at 298.15 K, we obtain

$$f(1.0\,\mathrm{mol\,L^{-1}} \to 2.0\,\mathrm{mol\,L^{-1}}) = \frac{\sigma(2.0\,\mathrm{mol\,L^{-1}}, 298.15\,\mathrm{K})}{\sigma(1.0\,\mathrm{mol\,L^{-1}}, 298.15\,\mathrm{K})} = \frac{1.31}{1.69} = 0.775,$$

and

$$f(1.0\,\mathrm{mol\,L^{-1}} \to 3.5\,\mathrm{mol\,L^{-1}}) = \frac{\sigma(3.0\,\mathrm{mol\,L^{-1}}, 298.15\,\mathrm{K})}{\sigma(1.0\,\mathrm{mol\,L^{-1}}, 298.15\,\mathrm{K})} = \frac{0.757}{1.69} = 0.448.$$

The two datasets overlap at approximately $c = 1.0\,\mathrm{mol\,L^{-1}}$ and $T = 298.15\,\mathrm{K}$, but give different values, namely $2.03\,\mathrm{S\,m^{-1}}$ and $1.69\,\mathrm{S\,m^{-1}}$. To obtain a reconciled reference value, their arithmetic mean was used,

$$\bar{\sigma}(1.0\,\mathrm{mol\,L^{-1}}, 298.15\,\mathrm{K}) = \frac{2.03 + 1.69}{2} = 1.86\,\mathrm{S\,m^{-1}}.$$

The reference conductivity at 333.15 K was then estimated as

$$\begin{aligned}\sigma_{\mathrm{ref}}(1.0\,\mathrm{mol\,L^{-1}}, 333.15\,\mathrm{K}) &= \bar{\sigma}(1.0\,\mathrm{mol\,L^{-1}}, 298.15\,\mathrm{K})\, f(298.15\,\mathrm{K} \to 333.15\,\mathrm{K}) \\ &= 1.86 \times 1.148 = 2.14\,\mathrm{S\,m^{-1}}.\end{aligned}$$

Reference values at higher concentrations were obtained by applying the concentration scaling factors,

$$\sigma_{\mathrm{ref}}(2.0\,\mathrm{mol\,L^{-1}}, 333.15\,\mathrm{K}) = 2.14 \times 0.775 = 1.66\,\mathrm{S\,m^{-1}},$$

$$\sigma_{\mathrm{ref}}(3.5\,\mathrm{mol\,L^{-1}}, 333.15\,\mathrm{K}) = 2.14 \times 0.448 = 0.96\,\mathrm{S\,m^{-1}}.$$

An uncertainty estimate was obtained from the mismatch between the two literature values at $c = 1.0\,\mathrm{mol\,L^{-1}}$ and $T = 298.15\,\mathrm{K}$. The sample standard deviation of the two values is

$$s = \sqrt{\frac{(2.04 - 1.86)^2 + (1.69 - 1.86)^2}{2 - 1}} = 0.248\,\mathrm{S\,m^{-1}},$$

corresponding to a relative inter-study scatter of

$$\frac{s}{\bar{\sigma}} = \frac{0.248}{1.86} = 0.133 \;\; (13.3\,\%).$$

This relative uncertainty was propagated to the reference conductivities at 333.15 K. The reference conductivities and uncertainties computed this way should be strictly interpreted as approximate benchmark values for the purpose of comparing the performance of the different molecular dynamics based conductivity estimators discussed in the paper. They do not represent reliable experimental measurements at the given conditions.

S5 Diffusion Coefficients

The self-diffusion coefficient D_k of a species k can be obtained using either the mean squared displacement (MSD) or the velocity autocorrelation function (VACF). In the Einstein formulation, the MSD of the species is computed and then a linear regression on the diffusive regime yields the

Table S3. Experimental conductivities of LiFSI/DME at different salt concentrations and temperatures sourced from literature, as well as calculated reference conductivities obtained from interpolation.

Source	c / mol L^{-1}	T / K	σ / S m^{-1}
Qian *et al.*[14]	1.0	298.15	1.69
	2.0	298.15	1.31
	3.0	298.15	0.94
	3.6	298.15	0.72
	4.0	298.15	0.57
	5.0	298.15	0.17
Zhao *et al.*[15]	1.0	253.15	1.17
	1.0	263.15	1.38
	1.0	273.15	1.62
	1.0	283.15	1.80
	1.0	293.15	1.96
	1.0	303.15	2.12
	1.0	313.15	2.26
	1.0	323.15	2.30
	1.0	333.15	2.33
Interpolation	1.0	333.15	2.14 ± 0.28
	2.0	333.15	1.66 ± 0.22
	3.5	333.15	0.96 ± 0.13

diffusion coefficient:

$$D_k = \frac{1}{6} \lim_{\tau \to \infty} \frac{\partial}{\partial \tau} \mathrm{MSD}_k\left(\tau\right), \tag{S1}$$

$$\mathrm{MSD}_k\left(\tau\right) = \left\langle \|\vec{r}_i(t+\tau) - \vec{r}_i(t)\|_2^2 \right\rangle_{t,i}, \tag{S2}$$

for all particles i of type k.

Using the VACF one obtains the Green-Kubo formulation for the diffusion coefficient:

$$D_k = \frac{1}{3} \int_0^\infty \langle \vec{v}_i(t) \cdot \vec{v}_i(0) \rangle_i dt. \tag{S3}$$

To estimate the magnitude of the finite-size effect on the self-diffusion coefficients, we additionally evaluated the Yeh–Hummer[16] correction for all systems,

$$\Delta D_{\mathrm{YH}} = \frac{2.837297\, k_{\mathrm{B}} T}{6 \pi \eta L}. \tag{S4}$$

For the [EMIm][DCA] systems, the viscosity at 353.15 K was estimated from experimental temperature-dependent datasets reported by Quijada-Maldonado *et al.*[17] and de Castro *et al.*[18], which were fitted to a VFT type equation and extrapolated to 353.15 K, yielding an estimate of

Table S4. Diffusion coefficients D, corresponding standard errors SE(D), Yeh–Hummer corrections ΔD_{YH}, and corrected diffusion coefficients $D_{\mathrm{corr}} = D + \Delta D_{\mathrm{YH}}$ in $10^{-10}\,\mathrm{m}^2\,\mathrm{s}^{-1}$ for all simulations of [EMIm][DCA].

Simulation	D	SE(D)	ΔD_{YH}	D_{corr}
		Anion		
IL^{125}	1.8807	0.0232	0.4813	2.3620
$\mathrm{IL}^{125}_{\mathrm{long}}$	1.9158	0.0185	0.4813	2.3971
IL^{250}	1.9755	0.0193	0.3820	2.3575
IL^{500}	2.0398	0.0140	0.3032	2.3430
IL^{1000}	2.0452	0.0124	0.2407	2.2859
$\mathrm{IL}^{1000}_{\mathrm{pol}}$	2.4142	0.0143	0.2407	2.6549
		Cation		
IL^{125}	1.6100	0.0218	0.4813	2.0913
$\mathrm{IL}^{125}_{\mathrm{long}}$	1.6297	0.0152	0.4813	2.1110
IL^{250}	1.6724	0.0131	0.3820	2.0544
IL^{500}	1.7255	0.0109	0.3032	2.0287
IL^{1000}	1.7747	0.0069	0.2407	2.0154
$\mathrm{IL}^{1000}_{\mathrm{pol}}$	2.1496	0.0099	0.2407	2.3903

($\eta \approx 4.7\,\mathrm{mPa\,s}$).

For the LiFSI/DME systems, direct viscosity data at the simulated temperatures and concentrations were not available. Therefore, viscosity estimates were constructed from interpolating the room-temperature values reported by Yin *et al.*[19] and applying a temperature correction from 298.15 K to 333.15 K based on neat 1,2-dimethoxyethane viscosity data reported by Zheng *et al.*[20] This procedure yielded viscosity estimates of 0.64 mPa s, 1.84 mPa s, and 8.94 mPa s for the 1.0 M, 2.0 M, and 3.5 M LiFSI/DME systems at 333.15 K, respectively.

The resulting Yeh–Hummer corrections and corrected diffusion coefficients are summarized in Tables S4 and S5.

Table S5. Diffusion coefficients D, corresponding standard errors $\mathrm{SE}(D)$, Yeh–Hummer corrections ΔD_{YH}, and corrected diffusion coefficients $D_{\mathrm{corr}} = D + \Delta D_{\mathrm{YH}}$ in $10^{-10}\,\mathrm{m^2\,s^{-1}}$ for all simulations of the LiFSI/DME system.

Simulation	D	SE(D)	ΔD_{YH}	D_{corr}
		Anion		
1.0 M	5.5465	0.0779	1.6974	7.2439
2.0 M	2.0822	0.0214	0.5967	2.6789
3.5 M	0.3030	0.0060	0.1241	0.4271
		Cation		
1.0 M	5.0220	0.0667	1.6974	6.7194
2.0 M	2.1117	0.0140	0.5967	2.7084
3.5 M	0.3503	0.0043	0.1241	0.4744
		Solvent		
1.0 M	10.4773	0.0449	1.6974	12.1747
2.0 M	4.5053	0.0109	0.5967	5.1020
3.5 M	0.9117	0.0134	0.1241	1.0358

S6 Single Trajectory Green-Kubo Calculations in TRAVIS

For the single replica case, TRAVIS assumes the last third of the running integral to be fluctuating about the constant plateau value. Under this assumption, TRAVIS chooses the average $\bar{I}$ of the integral values in this region $\{I_1, ..., I_n\}$ as the estimated plateau value. However, for large times, the correlation function shows significant noise, making the integral fluctuate strongly without clear convergence ("long-time noise problem"). Therefore, it is necessary to assess the uncertainty of this simple estimator accurately. To this extent, the integral values I_t are reformulated in terms of finite differences

$$I_t = I_1 + \sum_{i=1}^{t-1} D_i, \quad D_i = I_{i+1} - I_i, \tag{S5}$$

with the average value

$$\bar{I} = \frac{1}{n}\sum_{t=1}^{n} I_t = I_1 + \sum_{i=1}^{n-1} w_i D_i, \quad w_i = \frac{n-i}{n}. \tag{S6}$$

Under the assumptions that I_1 is a constant offset and the increments D_i are second-order stationary over the chosen region, the variance of the average can be written as

$$\mathrm{Var}(\bar{I}) = \sum_{i,j}^{n-1} w_i w_j \mathrm{Cov}(D_i, D_j) = \sum_{i,j}^{n-1} w_i w_j \gamma_D(i-j), \tag{S7}$$

with

$$\gamma_D(h) = \frac{1}{n-1} \sum_{\mathrm{i=1}}^{\mathrm{n-|h|-1}} (D_i - \bar{D})(D_{i+|h|} - \bar{D}), \tag{S8}$$

where γ_D is the estimated sample autocovariance of the finite differences D_i and $\mathrm{Cov}(D_i, D_j)$ as assumed to only depend on the lag $h = i - j$. Exploiting the symmetry of the autocovariance and by reordering the double-sum, one arrives at

$$\mathrm{Var}(\bar{I}) = \gamma_D(0) \sum_{i=1}^{n-1} w_i^2 + 2 \sum_{h=1}^{n-2} \gamma_D(h) c_h \quad \text{with} \quad c_h = \sum_{i=1}^{n-h-1} w_i w_{i+h}, \tag{S9}$$

which can be used to accurately obtain the standard-error by taking the square-root of the obtained variance.

S7 Additional Results

S7.1 Total Conductivities

Table S6. Ionic conductivities in $\mathrm{S\,m^{-1}}$ as obtained from different [EMIm][DCA] simulations with the Einstein–Helfand (EH) and Green–Kubo (GK) approach, as well as the corresponding Nernst–Einstein (NE) conductivities.

method	EH	GK	EH-NE	GK-NE
IL^{125}	5.184 ± 0.451	4.910 ± 1.800	6.820 ± 0.045	7.658 ± 0.533
$\mathrm{IL}^{125}_{\mathrm{long}}$	4.934 ± 0.255	4.683 ± 1.421	6.793 ± 0.029	7.004 ± 0.223
IL^{250}	4.753 ± 0.412	4.426 ± 1.282	7.011 ± 0.034	7.957 ± 0.376
IL^{500}	4.473 ± 0.356	4.803 ± 1.119	7.230 ± 0.017	7.256 ± 0.301
IL^{1000}	5.163 ± 0.274	5.692 ± 1.062	7.350 ± 0.022	7.976 ± 0.282
$\mathrm{IL}^{1000}_{\mathrm{pol}}$	4.779 ± 0.219	5.728 ± 1.855	8.820 ± 0.019	9.254 ± 0.241

Table S7. Ionic conductivities in $\mathrm{S\,m^{-1}}$ as obtained from LiFSI/DME simulations at different concentrations with the Einstein–Helfand (EH) and Green–Kubo (GK) approach, as well as the corresponding Nernst–Einstein (NE) conductivities.

method	EH	GK	EH-NE	GK-NE
1.0 M	2.118 ± 0.201	3.073 ± 0.729	3.473 ± 0.026	3.943 ± 0.132
2.0 M	1.350 ± 0.091	1.627 ± 0.654	2.809 ± 0.013	3.807 ± 0.120
3.5 M	0.420 ± 0.021	0.902 ± 0.243	0.789 ± 0.008	1.469 ± 0.128

S7.2 Inverse Haven-Ratio - Ionicities

In order to better capture the relationship between self- and collective transport, Figure S1 presents the inverse haven ratios of both investigated systems. Since they represent the ratio of collective conductivity and Nernst–Einstein conductivity, they specify the amount of the available self-diffusion that actually translates into effective charge transport. Values close to unity would

hint at mostly uncorrelated ion transport, whereas smaller values would imply a strong reduction due to ion-ion correlations.

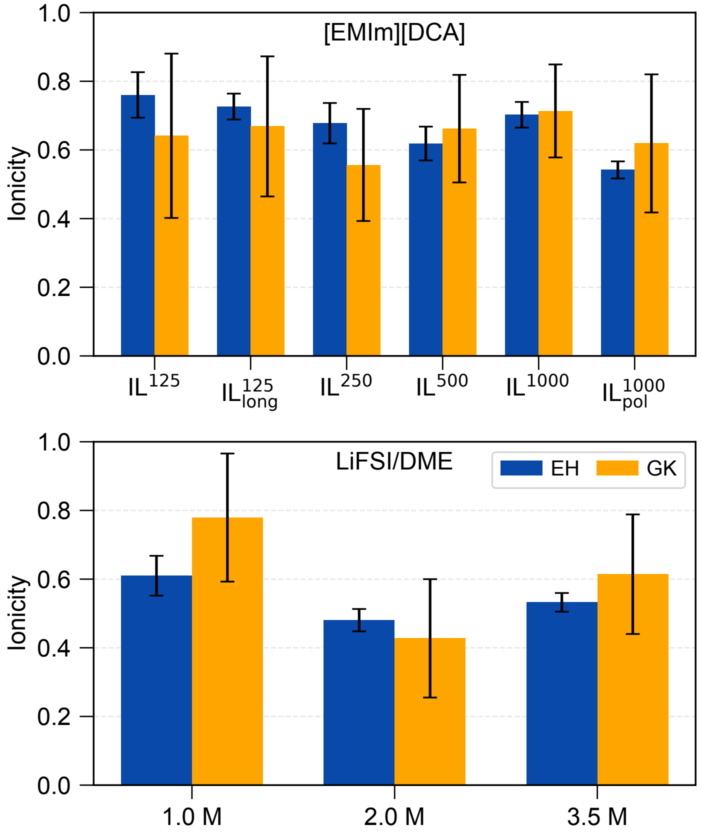

Figure S1. Calculated ionicities for all simulations of (top) [EMIm][DCA] and (bottom) LiFSI/DME. The blue bars denote ionicities obtained via the Einstein formulation, while the orange bars correspond to the ionicities from Green–Kubo-based correlation functions.

For the ionic liquid system, all ionicities are well below one.[21] The importance of correlation effects, already seen in the investigation of NE and collective conductivities, is therefore further examined. Within the CL&P series, the EH-based ionicities vary with system size. However, they do not show a clear trend but rather fluctuate within a narrow region between 0.62 and 0.76.[21] This hints at the fact that the degree of non-ideality does not change substantially despite of remaining finite size effects. One could therefore conclude that size effects do not influence the qualitative ratio of self- and collective transport, however, the shown standard errors suggest that even more sampling is needed to increase confidence.

A significant difference can be observed when comparing the IL^{1000} with the $\mathrm{IL}^{1000}_{\mathrm{pol}}$ simulations. Although the polarizable model shows larger self-diffusion coefficients, it exhibits substantially smaller ionicities (0.54 ± 0.02 *vs.* 0.70 ± 0.04). Thus, in the CL&Pol model, the gain in single-particle mobility is not proportionally translated into effective charge transport. In other words: the increased ion dynamics is accompanied by stronger or more consequential correlation effects.

In comparison to the quantities based on self-diffusion, like Nernst–Einstein conductivities or ideal transport numbers, the standard errors of the ionicities are visibly larger. This is reasonable because the ionicity is directly proportional to the collective conductivity and is therefore dependent on the statistically demanding contributions that contain ion-ion cross correlations. Consequently, the increased uncertainties are not surprising, but reflect that the collective transport quantities obtained from equilibrium simulations are generally more sensitive to sampling and noise.[22]

However, GK-based ionicities show the same overall behavior, with significantly larger standard errors (from about 0.13 to 0.24). Although the mean values are comparable to those obtained from the EH approach, differences between these values cannot be reliably resolved due to the large uncertainties.

Also, for the salt-in-solvent system, the calculated ionicities are consistently far below unity for all concentrations.[23] Therefore, here as well, the Nernst–Einstein approximation overestimates the actual charge transport systematically, and collective ion movement stays relevant over the whole concentration range.

Within the EH-based values, a clear decrease in ionicity can be observed from 0.61 at 1.0 M to approximately 0.48 at 2.0 M. Moving to even higher concentration, the ionicity shows a slight increase to about 0.53 at 3.5 M. The non-ideality of charge transport seems to be most pronounced at the intermediate concentration. However, the increase in ionicity from 2.0 to 3.5 M can only be substantiated to a limited extent, compared to the difference between the two lower concentrations. Due to the non-negligible standard errors, the data show a tendency for the re-increase of the ionicity, rather than a cleanly resolved trend. Physically, this behavior suggests that the degree of non-ideality does not just monotonically increase with salt concentration, but reacts sensitively to the interplay of solvation, ion-association and collective movement. Especially in concentrated electrolytes it is reasonable that these influences do not change linearly with concentration. Rather different local transport mechanisms dominate at certain concentration ranges and thus influence the amount and impact of ion correlation on the effective charge transport.[24]

The GK-based ionicities follow the same general trend, although they carry significantly larger standard errors. Although the mean values qualitatively agree with the EH-results, the uncertainties are too large to quantitatively assess the concentration dependence. As far conductivities and ideal transport numbers, the EH-based values show a cleaner and statistically more robust picture.

In total, the ionicities summarize the previously discussed differences in self and collective ion transport in one single dimensionless quantity. For both systems it is evident that a significant part of the single-particle mobility does not contribute to the macroscopic charge transport. Therefore, ionicities represent a useful quantity for the evaluation of the importance of correlation effects in ionic systems, independent of the absolute conductivities. However, even for the presented EH-analysis, which enables a substantially more robust assessment than GK, extensive sampling is required for the reliable resolution of subtle differences.

Table S8. Ionicities of the [EMIm][DCA] systems obtained from the EH and GK approaches.

System	EH	GK
IL^{125}	0.760 ± 0.066	0.641 ± 0.239
IL^{125}_{long}	0.726 ± 0.038	0.669 ± 0.204
IL^{250}	0.678 ± 0.059	0.556 ± 0.163
IL^{500}	0.619 ± 0.049	0.662 ± 0.157
IL^{1000}	0.702 ± 0.037	0.714 ± 0.135
IL^{1000}_{pol}	0.542 ± 0.025	0.619 ± 0.201

Table S9. Ionicities of the LiFSI/DME systems obtained from the EH and GK approaches.

System	EH	GK
1.0 M	0.610 ± 0.058	0.779 ± 0.187
2.0 M	0.480 ± 0.033	0.427 ± 0.172
3.5 M	0.532 ± 0.027	0.614 ± 0.174

S7.3 Charge Current Autocorrelation Functions

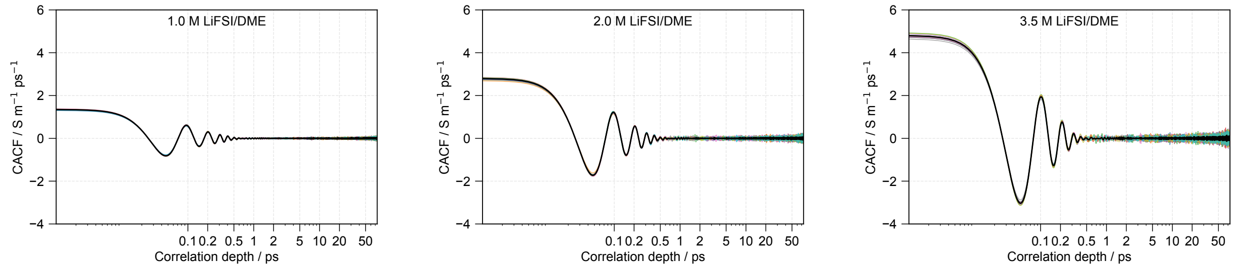

Figure S2. Charge autocorrelation functions (CACFs) of LiFSI/DME at different salt concentrations. The black curve shows the averaged CACF. The colored curves show the individual replica CACFs.

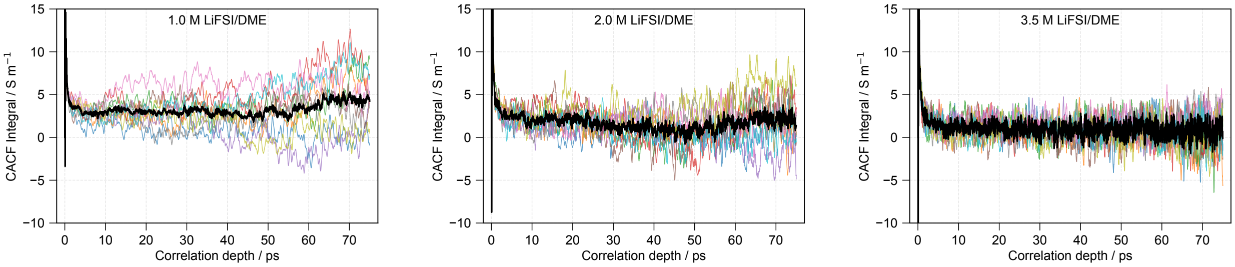

Figure S3. CACF integrals of the LiFSI/DME system at different salt concentrations. The black curve shows the integral of the averaged CACF. The colored curves show 200 fs moving averages of the individual replica integrals.

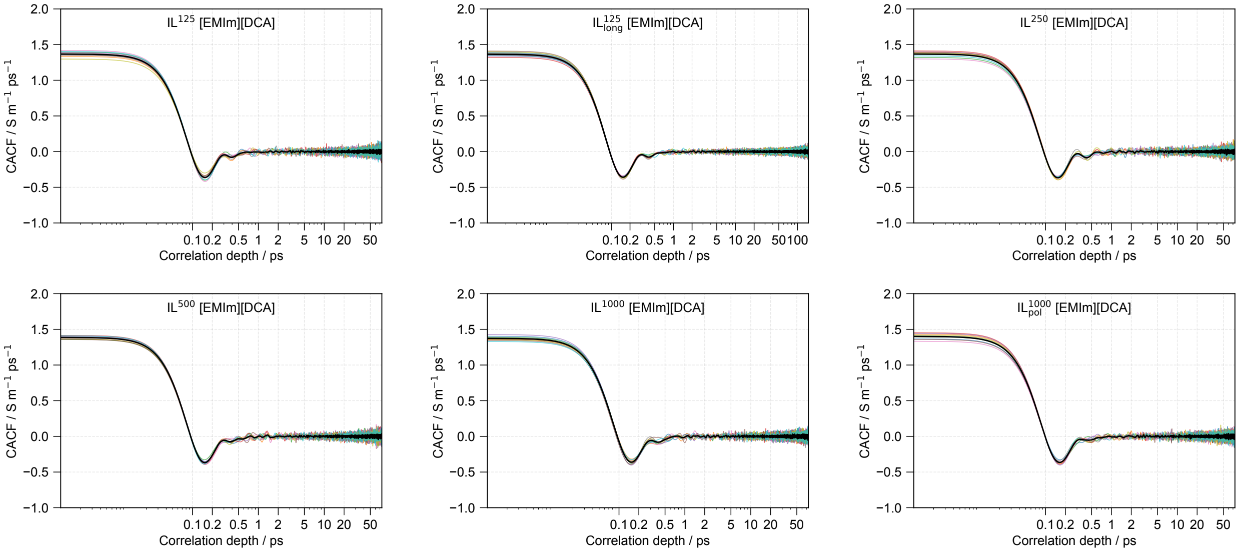

Figure S4. Charge autocorrelation functions (CACFs) as computed from different [EMIm][DCA] simulations. The black curve shows the averaged CACF. The colored curves show the individual replica CACFs.

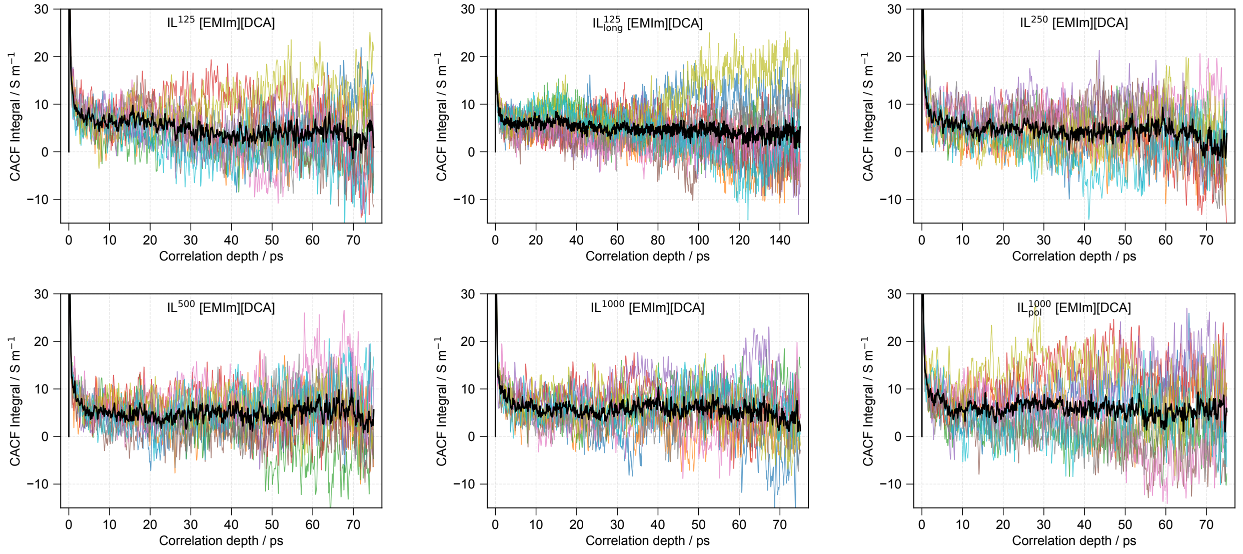

Figure S5. CACF integrals as computed from different [EMIm][DCA] simulations. The black curve shows the integral of the averaged CACF. The colored curves show 200 fs moving averages of the individual replica integrals.

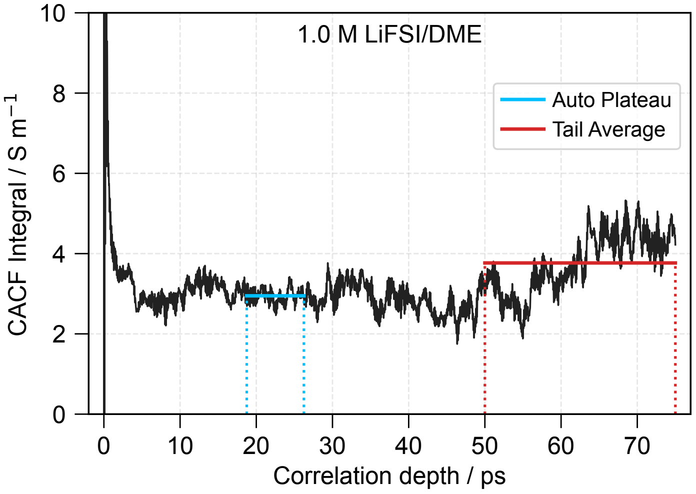

Figure S6. Comparison of plateau identification methods applied to the average CACF's integral as obtained from the LiFSI/DME simulations at a salt concentration of 1.0 M. The automated plateau search identifies a window of 18.75 ps to 26.25 ps, yielding a conductivity estimate of 2.95 $\mathrm{S\,m^{-1}}$. Treating instead the CACF integral tail from 50 ps to 75 ps as plateau window yields an estimate of 3.77 $\mathrm{S\,m^{-1}}$, significantly overestimating the reference value of 2.14 $\mathrm{S\,m^{-1}}$.

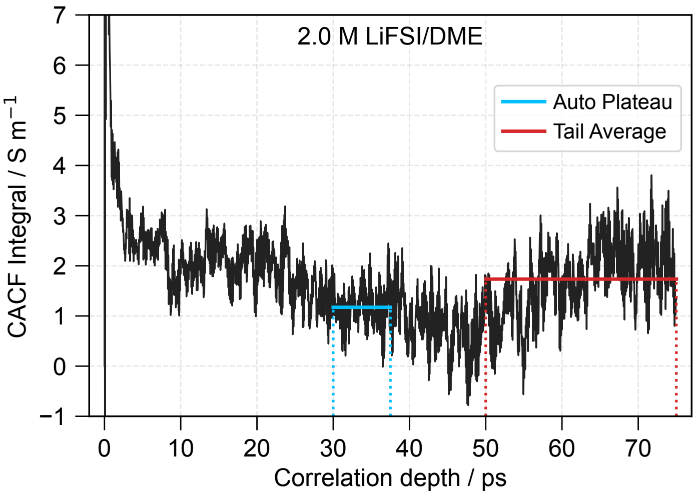

Figure S7. Comparison of plateau identification methods applied to the average CACF's integral as obtained from the LiFSI/DME simulations at a salt concentration of 2.0 M. The automated plateau search identifies a window of 30.00 ps to 37.50 ps, yielding a conductivity estimate of 1.17 $\mathrm{S\,m^{-1}}$. Treating instead the CACF integral tail from 50 ps to 75 ps as plateau window yields an estimate of 1.73 $\mathrm{S\,m^{-1}}$.

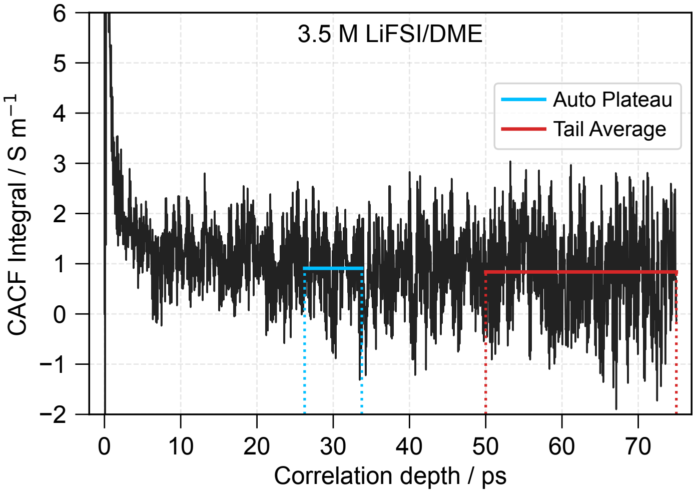

Figure S8. Comparison of plateau identification methods applied to the average CACF's integral as obtained from the LiFSI/DME simulations at a salt concentration of 3.5 M. The automated plateau search identifies a window of 26.25 ps to 33.75 ps, yielding a conductivity estimate of 0.91 S m^{-1}. Treating instead the CACF integral tail from 50 ps to 75 ps as plateau window yields an estimate of 0.84 S m^{-1}.

S7.4 Diffusion coefficients

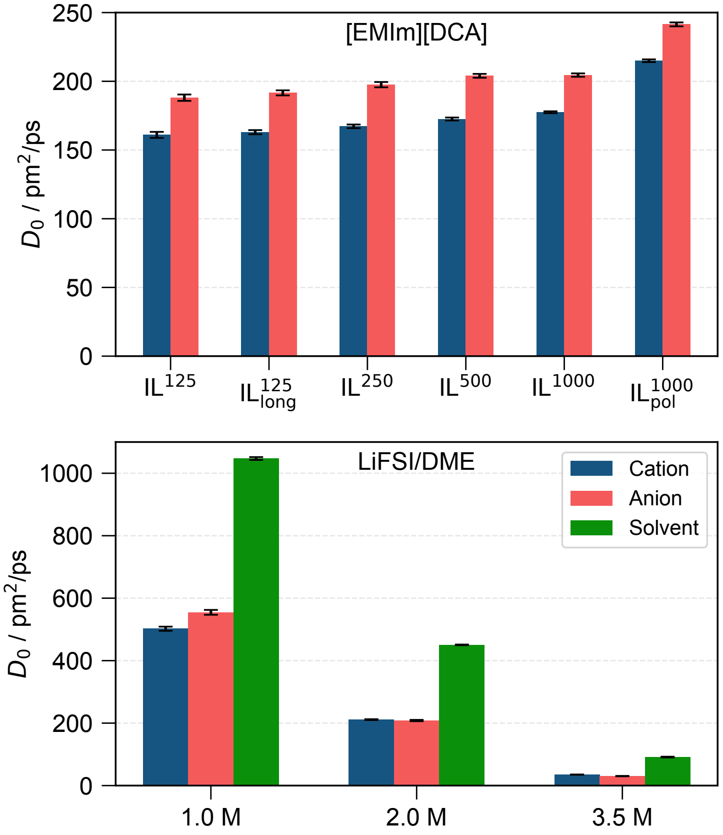

Figure S9. Self-diffusion coefficients of the different simulated systems as obtained from molecular dynamics simulations.

S7.5 Collective MSD

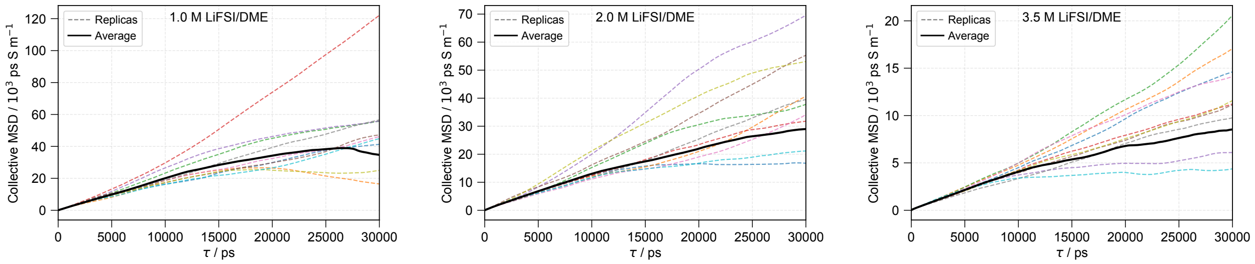

Figure S10. Collective mean square displacements (CMSDs) of LiFSI/DME at different salt concentrations. The black curve shows the averaged CMSD, and the colored curves show the individual replica CMSDs.

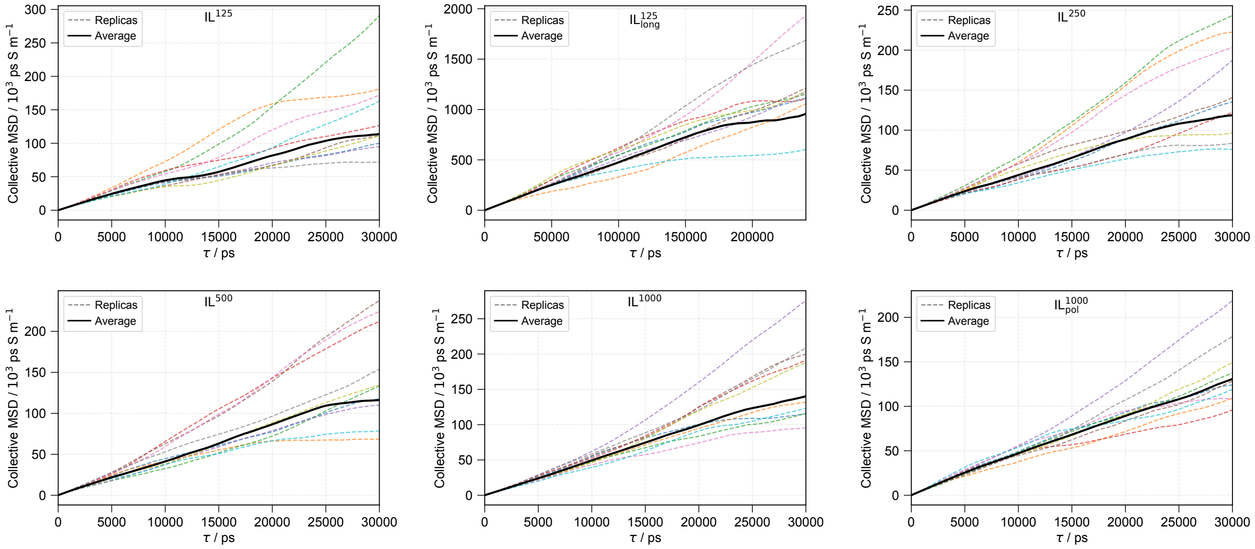

Figure S11. Collective mean square displacements (CMSDs) as computed from different [EMIm][DCA] simulations. The black curve shows the averaged CMSD, and the colored curves show the individual replica CMSDs.

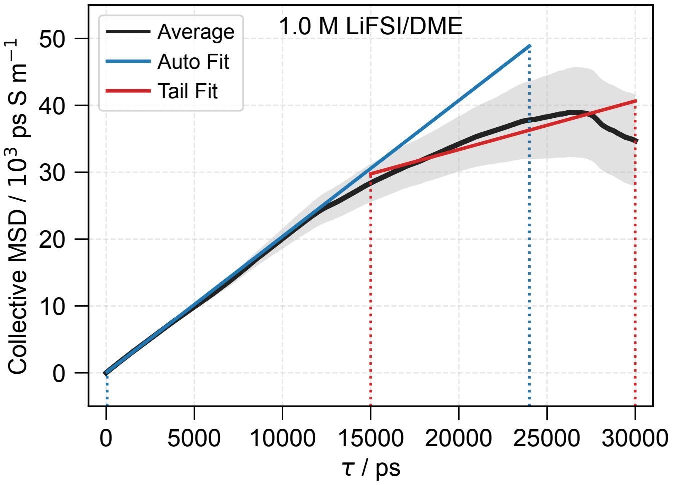

Figure S12. Comparison of fitting methods applied to the average CMSD as obtained from the LiFSI/DME simulations at a salt concentration of 1.0 M. The automated fit window search as implemented in TRAVIS identifies a window of 60 ps to 24 ns as diffusive regime. The weighted fit emphasizes the early to mid-time regions with low uncertainties, yielding a conductivity estimate of 2.03 S m^{-1} close to the reference value of 2.14 S m^{-1}. The fixed tail fit treats the second half of the CMSD, 15 ns to 30 ns, as fit window. The weighted fit in that range yields an estimate of 0.73 S m^{-1}, significantly underestimating the reference value.

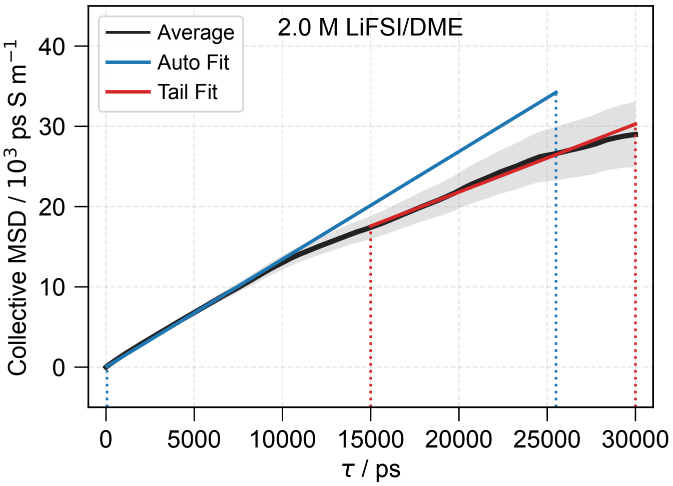

Figure S13. Comparison of fitting methods applied to the average CMSD as obtained from the LiFSI/DME simulations at a salt concentration of 2.0 M. The automated fit window search as implemented in TRAVIS identifies a window of 60 ps to 25.5 ns as diffusive regime. The weighted fit emphasizes the early to mid-time regions with low uncertainties, yielding a conductivity estimate of 1.34 S m^{-1}. The fixed tail fit treats the second half of the CMSD, 15 ns to 30 ns, as fit window. The weighted fit in that range yields an estimate of 0.85 S m^{-1}.

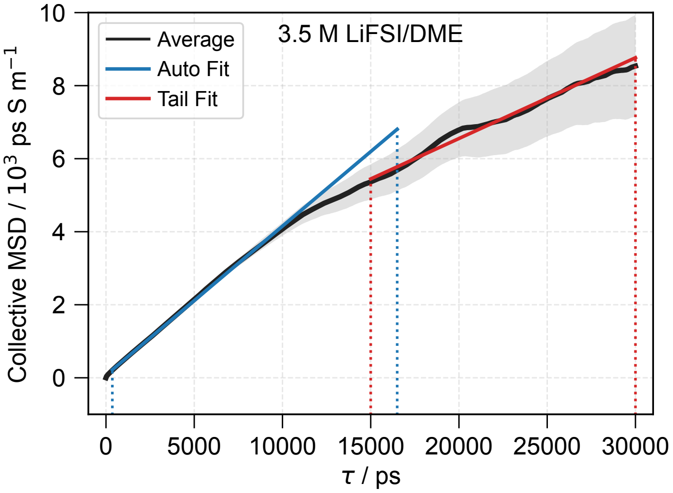

Figure S14. Comparison of fitting methods applied to the average CMSD as obtained from the LiFSI/DME simulations at a salt concentration of 3.5 M. The automated fit window search as implemented in TRAVIS identifies a window of 360 ps to 16.5 ns as diffusive regime. The weighted fit emphasizes the early to mid-time regions with low uncertainties, yielding a conductivity estimate of $0.41\,\mathrm{S\,m^{-1}}$. The fixed tail fit treats the second half of the CMSD, 15 ns to 30 ns, as fit window. The weighted fit in that range yields an estimate of $0.22\,\mathrm{S\,m^{-1}}$.

S7.6 Transport Numbers

Table S10. Ideal transport numbers of the [EMIm][DCA] systems obtained from the EH and GK approaches, resolved into cation and anion self contributions.

System	EH cation	EH anion	GK cation	GK anion
IL^{125}	0.461 ± 0.003	0.539 ± 0.003	0.467 ± 0.035	0.533 ± 0.035
IL^{125}_{long}	0.458 ± 0.002	0.542 ± 0.002	0.470 ± 0.016	0.530 ± 0.016
IL^{250}	0.460 ± 0.002	0.540 ± 0.002	0.466 ± 0.024	0.534 ± 0.024
IL^{500}	0.461 ± 0.001	0.539 ± 0.001	0.471 ± 0.020	0.529 ± 0.020
IL^{1000}	0.464 ± 0.001	0.536 ± 0.001	0.463 ± 0.018	0.537 ± 0.018
IL^{1000}_{pol}	0.471 ± 0.001	0.529 ± 0.001	0.467 ± 0.013	0.533 ± 0.013

Table S11. Ideal transport numbers of the LiFSI/DME systems obtained from the EH and GK approaches, resolved into cation and anion self contributions.

System	EH cation	EH anion	GK cation	GK anion
1.0 M	0.478 ± 0.003	0.522 ± 0.003	0.479 ± 0.016	0.521 ± 0.016
2.0 M	0.503 ± 0.002	0.497 ± 0.002	0.481 ± 0.016	0.519 ± 0.016
3.5 M	0.532 ± 0.004	0.468 ± 0.004	0.509 ± 0.044	0.491 ± 0.044

Table S12. Real transport numbers of the [EMIm][DCA] systems in the mass-fixed reference frame, obtained from the EH and GK approaches.

System	EH cation	EH anion	GK cation	GK anion
IL^{125}	0.373 ± 0.038	0.627 ± 0.038	0.372 ± 0.159	0.628 ± 0.159
IL^{125}_{long}	0.371 ± 0.022	0.629 ± 0.022	0.372 ± 0.131	0.628 ± 0.131
IL^{250}	0.373 ± 0.038	0.627 ± 0.038	0.370 ± 0.122	0.630 ± 0.122
IL^{500}	0.373 ± 0.034	0.627 ± 0.034	0.373 ± 0.100	0.627 ± 0.100
IL^{1000}	0.373 ± 0.023	0.627 ± 0.023	0.371 ± 0.080	0.629 ± 0.080
IL^{1000}_{pol}	0.373 ± 0.020	0.627 ± 0.020	0.372 ± 0.140	0.628 ± 0.140

Table S13. Real transport numbers of the LiFSI/DME systems in different reference frames, obtained from the EH and GK approaches.

Frame	System	EH cation	EH anion	GK cation	GK anion
Mass-fixed	1.0 M	0.439 ± 0.050	0.561 ± 0.050	0.484 ± 0.145	0.516 ± 0.145
	2.0 M	0.451 ± 0.046	0.549 ± 0.046	0.443 ± 0.234	0.557 ± 0.234
	3.5 M	0.373 ± 0.062	0.627 ± 0.062	0.643 ± 0.224	0.357 ± 0.224
Number-fixed	1.0 M	0.355 ± 0.052	0.645 ± 0.052	0.396 ± 0.147	0.604 ± 0.147
	2.0 M	0.289 ± 0.049	0.711 ± 0.049	0.275 ± 0.253	0.725 ± 0.253
	3.5 M	0.168 ± 0.065	0.832 ± 0.065	0.396 ± 0.227	0.604 ± 0.227
Solvent-fixed	1.0 M	0.320 ± 0.061	0.680 ± 0.061	0.378 ± 0.170	0.622 ± 0.170
	2.0 M	0.201 ± 0.097	0.799 ± 0.097	0.163 ± 0.378	0.837 ± 0.378
	3.5 M	-0.174 ± 0.278	1.174 ± 0.278	0.286 ± 0.560	0.714 ± 0.560

S7.7 Replica vs Segments

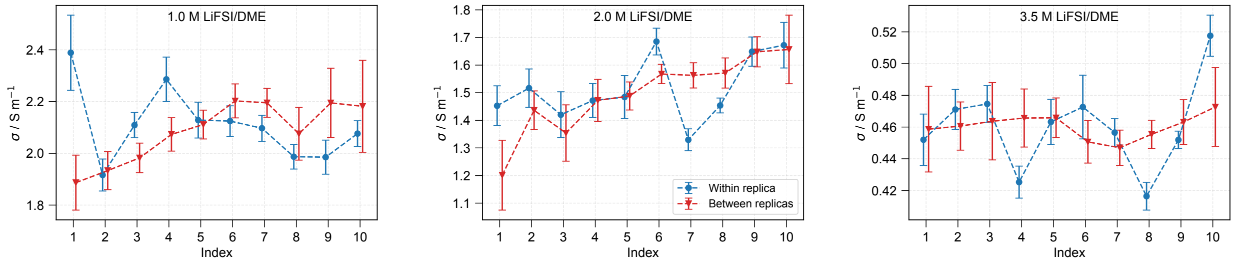

Figure S15. Comparison of conductivity estimates obtained from averaging within replicas and between replicas for the LiFSI/DME system at different concentrations. The index on the x-axis denotes the replica or segment index, respectively.

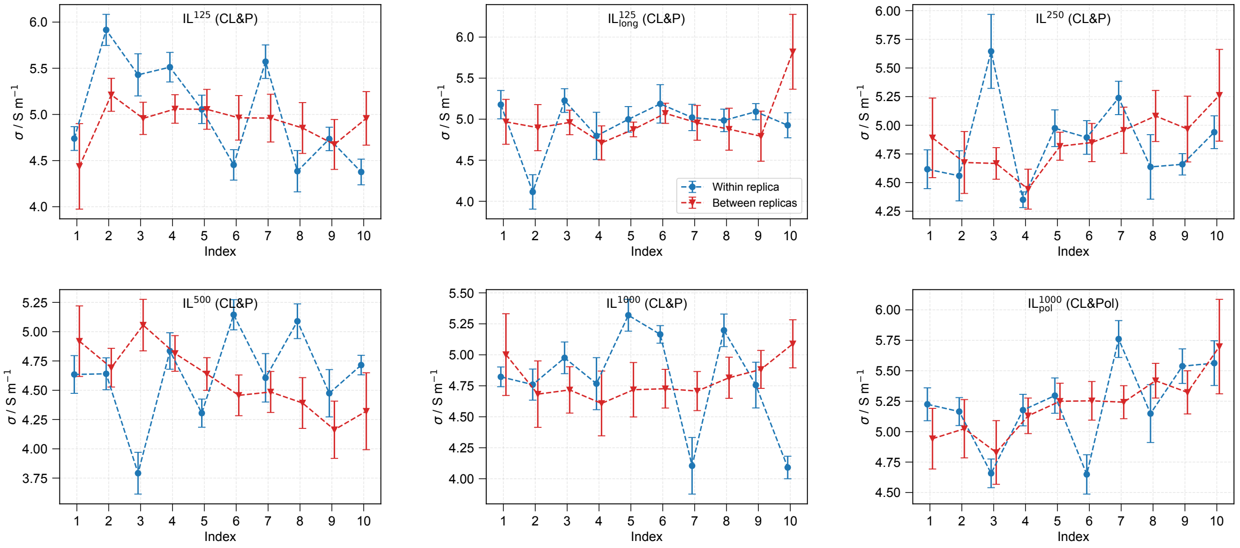

Figure S16. Comparison of conductivity estimates obtained from averaging within replicas and between replicas for the different [EMIm][DCA] simulations. The index on the x-axis denotes the replica or segment index, respectively.

S7.8 Correlation Depth

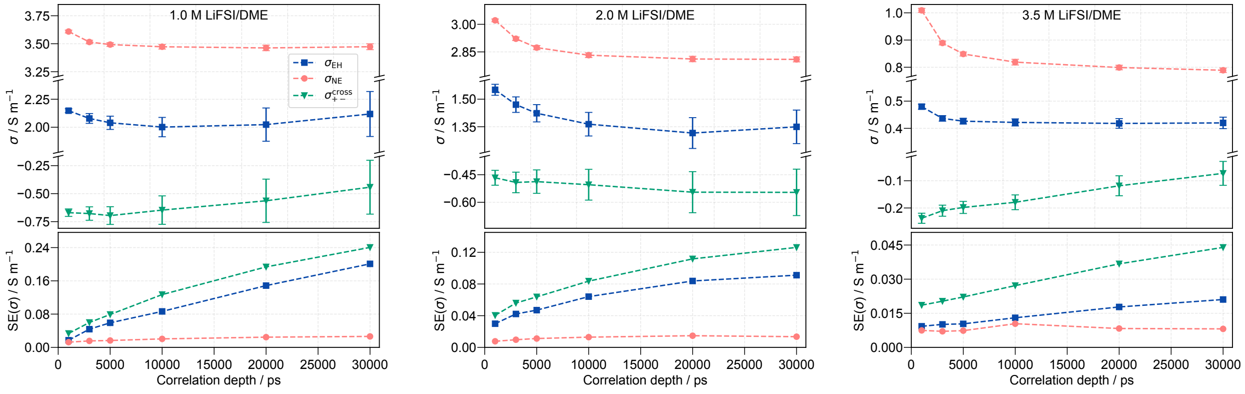

Figure S17. EH conductivity, NE conductivity, and anion-cation cross contribution as obtained from the LiFSI/DME simulations with different maximum correlation depths (top) and the corresponding standard error (bottom).

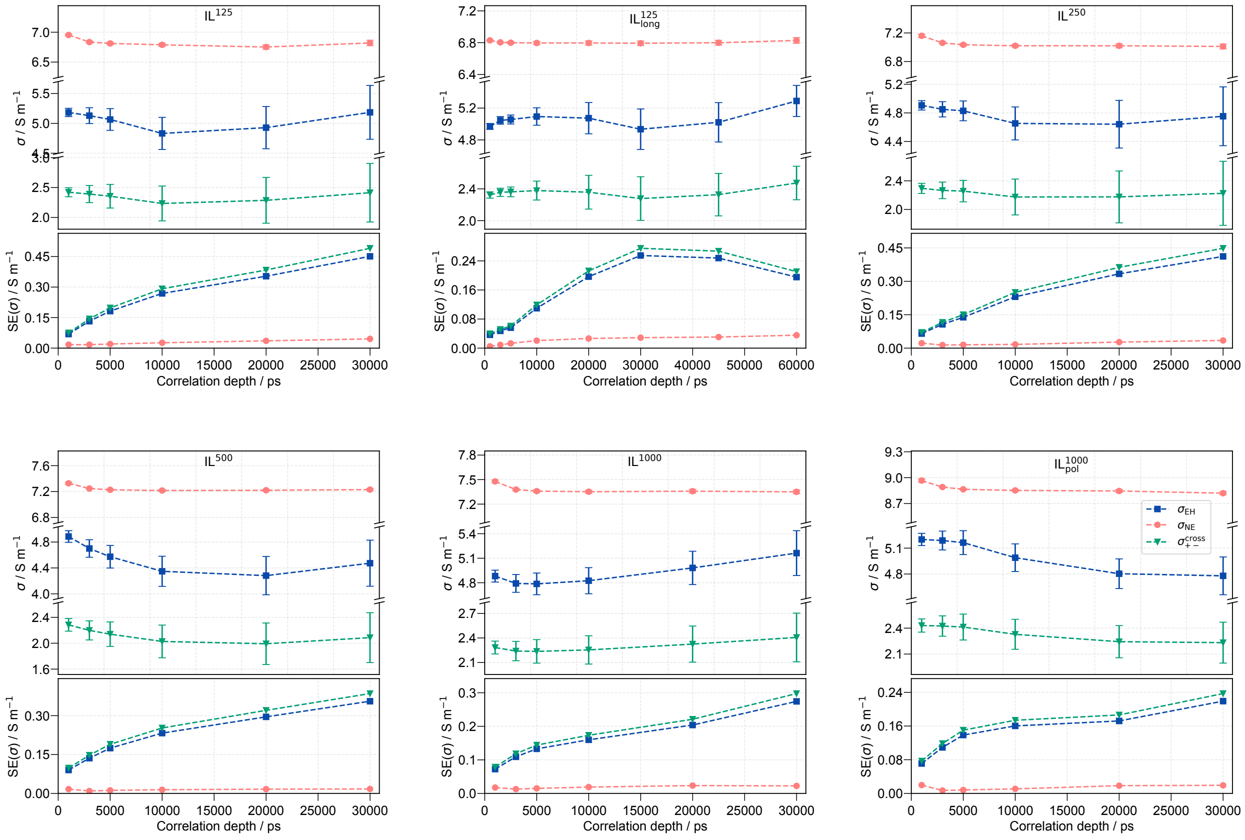

Figure S18. EH conductivity, NE conductivity, and anion-cation cross contribution as obtained from the [EMIm][DCA] simulations with different maximum correlation depths (top) and the corresponding standard error (bottom).

S7.9 Decomposition

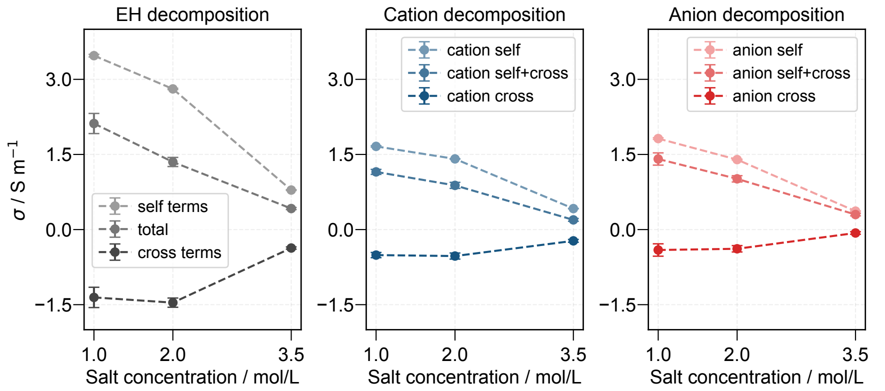

Figure S19. Decomposition into self- and cross-terms of the (left) total Einstein–Helfand conductivity, (center) the cation contributions, and (right) the anion contributions, as obtained from the LiFSI/DME simulations at different salt concentrations.

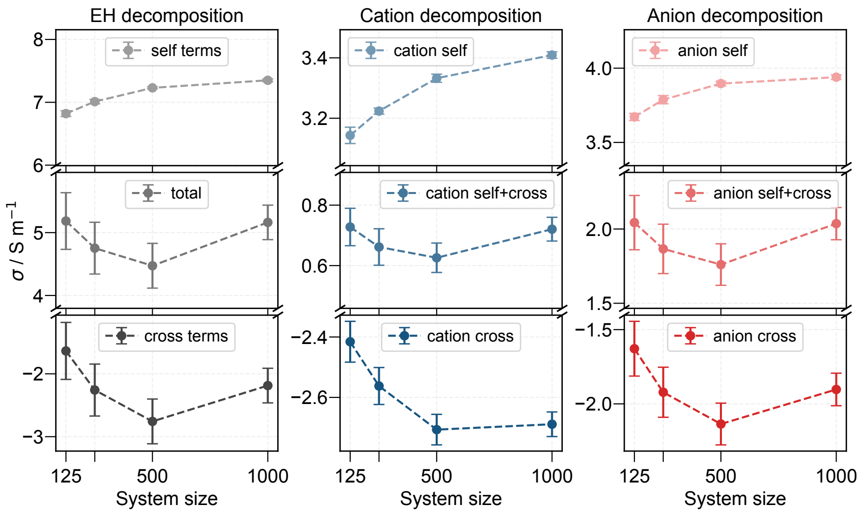

Figure S20. Decomposition into self- and cross-terms of the (left) total Einstein–Helfand conductivity, (center) the cation contributions, and (right) the anion contributions, as obtained from the CL&P simulations of [EMIm][DCA] containing different amounts of ion pairs.

Table S14. Total Einstein–Helfand conductivity decomposition of the [EMIm][DCA] systems. All values are given in $\mathrm{S\,m^{-1}}$.

System	Total	Self terms	Cross terms
IL^{125}	5.184 ± 0.451	6.820 ± 0.045	-1.636 ± 0.453
$\mathrm{IL}^{125}_{\mathrm{long}}$	4.934 ± 0.255	6.793 ± 0.029	-1.859 ± 0.256
IL^{250}	4.753 ± 0.412	7.011 ± 0.034	-2.258 ± 0.413
IL^{500}	4.473 ± 0.356	7.230 ± 0.017	-2.757 ± 0.356
IL^{1000}	5.163 ± 0.274	7.350 ± 0.022	-2.187 ± 0.275
$\mathrm{IL}^{1000}_{\mathrm{pol}}$	4.779 ± 0.219	8.820 ± 0.019	-4.042 ± 0.220

Table S15. Ion-resolved Einstein–Helfand conductivity decomposition of the [EMIm][DCA] systems in the barycentric reference frame. All values are given in $\mathrm{S\,m^{-1}}$.

System	Cation self	Cation cross	Anion self	Anion cross	Anion-cation
IL^{125}	3.143 ± 0.027	-2.416 ± 0.068	3.672 ± 0.024	-1.629 ± 0.184	2.413 ± 0.491
$\mathrm{IL}^{125}_{\mathrm{long}}$	3.112 ± 0.014	-2.418 ± 0.037	3.679 ± 0.025	-1.717 ± 0.100	2.278 ± 0.275
IL^{250}	3.224 ± 0.010	-2.562 ± 0.061	3.788 ± 0.028	-1.922 ± 0.169	2.225 ± 0.448
IL^{500}	3.332 ± 0.013	-2.707 ± 0.051	3.896 ± 0.016	-2.136 ± 0.141	2.086 ± 0.385
IL^{1000}	3.409 ± 0.011	-2.689 ± 0.041	3.939 ± 0.015	-1.903 ± 0.110	2.406 ± 0.298
$\mathrm{IL}^{1000}_{\mathrm{pol}}$	4.157 ± 0.010	-3.490 ± 0.032	4.661 ± 0.016	-2.781 ± 0.087	2.232 ± 0.237

Table S16. Total Green–Kubo conductivity decomposition of the [EMIm][DCA] systems. All values are given in $\mathrm{S\,m^{-1}}$.

System	Total	Self terms	Cross terms
IL^{125}	4.910 ± 1.800	7.658 ± 0.533	-2.748 ± 1.878
$\mathrm{IL}^{125}_{\mathrm{long}}$	4.683 ± 1.421	7.004 ± 0.223	-2.322 ± 1.439
IL^{250}	4.426 ± 1.282	7.957 ± 0.376	-3.531 ± 1.336
IL^{500}	4.803 ± 1.119	7.256 ± 0.301	-2.453 ± 1.159
IL^{1000}	5.692 ± 1.062	7.976 ± 0.282	-2.285 ± 1.098
$\mathrm{IL}^{1000}_{\mathrm{pol}}$	5.728 ± 1.855	9.254 ± 0.241	-3.526 ± 1.871

Table S17. Ion-resolved Gree–Kubo conductivity decomposition of the [EMIm][DCA] systems in the barycentric reference frame. All values are given in $\mathrm{S\,m^{-1}}$.

System	Cation self	Cation cross	Anion self	Anion cross	Anion-cation
IL^{125}	3.575 ± 0.371	-2.913 ± 0.450	4.083 ± 0.383	-2.913 ± 0.450	2.327 ± 1.955
$\mathrm{IL}^{125}_{\mathrm{long}}$	3.293 ± 0.143	-2.641 ± 0.243	3.712 ± 0.172	-2.641 ± 0.243	2.177 ± 1.539
IL^{250}	3.705 ± 0.259	-3.091 ± 0.303	4.252 ± 0.273	-3.091 ± 0.303	2.049 ± 1.373
IL^{500}	3.420 ± 0.175	-2.752 ± 0.227	3.836 ± 0.244	-2.752 ± 0.227	2.246 ± 1.210
IL^{1000}	3.696 ± 0.200	-2.902 ± 0.248	4.280 ± 0.198	-2.902 ± 0.248	2.630 ± 1.149
$\mathrm{IL}^{1000}_{\mathrm{pol}}$	4.324 ± 0.126	-3.526 ± 0.287	4.931 ± 0.205	-2.661 ± 0.767	2.661 ± 2.014

Table S18. Total Einstein–Helfand conductivity decomposition of the LiFSI/DME systems. All values are given in $\mathrm{S\,m^{-1}}$.

System	Total	Self terms	Cross terms
1.0 M	2.118 ± 0.201	3.473 ± 0.026	-1.355 ± 0.203
2.0 M	1.350 ± 0.091	2.809 ± 0.013	-1.460 ± 0.092
3.5 M	0.420 ± 0.021	0.789 ± 0.008	-0.369 ± 0.023

Table S19. Ion-resolved Einstein–Helfand conductivity decomposition of the LiFSI/DME systems in different reference frames. All values are given in $\mathrm{S\,m^{-1}}$.

System	Cation self	Cation cross	Anion self	Anion cross	Anion-cation
**Mass-fixed**					
1.0 M	1.661 ± 0.014	-0.510 ± 0.052	1.817 ± 0.017	-0.408 ± 0.123	-0.443 ± 0.240
2.0 M	1.411 ± 0.006	-0.529 ± 0.061	1.397 ± 0.009	-0.384 ± 0.064	-0.546 ± 0.126
3.5 M	0.419 ± 0.004	-0.226 ± 0.029	0.368 ± 0.005	-0.069 ± 0.027	-0.073 ± 0.044
**Number-fixed**					
1.0 M	1.661 ± 0.014	-0.646 ± 0.050	1.819 ± 0.017	-0.189 ± 0.147	-0.527 ± 0.253
2.0 M	1.410 ± 0.006	-0.667 ± 0.054	1.398 ± 0.009	-0.084 ± 0.077	-0.708 ± 0.130
3.5 M	0.419 ± 0.004	-0.251 ± 0.023	0.369 ± 0.005	0.078 ± 0.033	-0.195 ± 0.045
**Solvent-fixed**					
1.0 M	1.663 ± 0.014	-0.325 ± 0.071	1.822 ± 0.017	0.279 ± 0.184	-1.322 ± 0.281
2.0 M	1.413 ± 0.006	0.066 ± 0.120	1.401 ± 0.009	0.886 ± 0.141	-2.417 ± 0.206
3.5 M	0.420 ± 0.004	0.230 ± 0.074	0.371 ± 0.005	0.845 ± 0.105	-1.445 ± 0.130

Table S20. Total Green–Kubo conductivity decomposition of the LiFSI/DME systems. All values are given in $\mathrm{S\,m^{-1}}$.

System	Total	Self terms	Cross terms
1.0 M	3.073 ± 0.729	1.956 ± 0.132	1.117 ± 0.740
2.0 M	1.627 ± 0.654	1.903 ± 0.120	-0.276 ± 0.665
3.5 M	0.902 ± 0.243	0.843 ± 0.128	0.059 ± 0.275

Table S21. Ion-resolved Green–Kubo conductivity decomposition of the LiFSI/DME systems in different reference frames. All values are given in $\mathrm{S\,m^{-1}}$.

System	Cation self	Cation cross	Anion self	Anion cross	Anion-cation
**Mass-fixed**					
1.0 M	1.889 ± 0.067	-0.521 ± 0.403	2.055 ± 0.113	-0.591 ± 0.431	0.242 ± 0.928
2.0 M	1.830 ± 0.074	-0.834 ± 0.276	1.977 ± 0.095	-0.796 ± 0.415	-0.549 ± 0.814
3.5 M	0.748 ± 0.095	-0.202 ± 0.193	0.720 ± 0.087	-0.432 ± 0.196	0.068 ± 0.344
**Number-fixed**					
1.0 M	1.887 ± 0.067	-0.739 ± 0.339	2.057 ± 0.112	-0.268 ± 0.517	0.136 ± 0.947
2.0 M	1.829 ± 0.074	-1.019 ± 0.218	1.978 ± 0.095	-0.434 ± 0.561	-0.727 ± 0.881
3.5 M	0.748 ± 0.094	-0.434 ± 0.155	0.720 ± 0.087	-0.220 ± 0.265	0.088 ± 0.370
**Solvent-fixed**					
1.0 M	1.889 ± 0.068	-0.456 ± 0.417	2.060 ± 0.112	0.124 ± 0.632	-0.544 ± 1.042
2.0 M	1.831 ± 0.074	-0.264 ± 0.373	1.982 ± 0.095	0.683 ± 0.921	-2.605 ± 1.184
3.5 M	0.748 ± 0.095	0.011 ± 0.439	0.721 ± 0.087	0.425 ± 0.714	-1.003 ± 0.863

S7.10 Discrepancy between EH and GK results - Additional Analyses

max. depth [ps]	σ^{EH}	σ^{GK}
100	4.8084	4.8802
400	5.2057	4.9556
1000	4.5776	4.0158

Table S22. Einstein–Helfand and Green–Kubo analyses of a 10 ns long trajectory of 1000 [EMIm][DCA] ion pairs with the CL&P force field and a dumping frequency of 20 fs for different maximum correlation depths. Both methods show consistent results over a significant correlation depth range. EH might suffer from inclusion of parts of the balistic regime, while GK might suffer from integral inaccuracies due to the comparably low dumping frequency. While this trajectory might offer a more reasonable comparison of the two methods, it requires over 900 GB of disc space, making it unaffordable to store multiple sets of replicas of similar simulations.

Method	σ [S m^{-1}]	SE [S m^{-1}]
**linear regime / plateau**		
EH	3.220	0.282
GK	3.073	0.729
**tail fit / average, unweighted**		
EH	3.637	1.078
GK	3.767	0.940

Table S23. Conductivities and corresponding standard errors obtained from the 10 short (100 ps) replicas of the 1.0 M LiFSI/DME system with different analysis methods. When using the same set of trajectories and similar cross-replica averaging schemes, the obtained conductivities are in excellent agreement within their standard errors.

References

[1] K. Goloviznina, J. N. Canongia Lopes, M. Costa Gomes, A. A. H. Páádua, *J. Chem. Theory Comput.* **2019**, *15*, 5858.

[2] K. Goloviznina, Z. Gong, A. A. H. Pádua, *WIREs Comput. Mol. Sci.* **2021**, *12*.

[3] A. Massaro, J. Avila, K. Goloviznina, I. Rivalta, C. Gerbaldi, M. Pavone, M. F. Costa Gomes, A. A. H. Padua, *Phys. Chem. Chem. Phys.* **2020**, *22*, 20114.

[4] K. Goloviznina, Z. Gong, M. F. Costa Gomes, A. A. H. Pádua, *J. Chem. Theory Comput.* **2021**, *17*, 1606.

[5] M. E. Di Pietro, K. Goloviznina, A. van den Bruinhorst, G. de Araujo Lima e Souza, M. Costa Gomes, A. A. H. Padua, A. Mele, *ACS Sustain. Chem. Eng.* **2022**, *10*, 11835.

[6] F. Philippi, D. Rauber, O. Palumbo, K. Goloviznina, J. McDaniel, D. Pugh, S. Suarez, C. C. Fraenza, A. Padua, C. W. M. Kay, T. Welton, *Chem. Sci.* **2022**, *13*, 9176.

[7] A. Padua, fftool v1.0.0 **2015**.

[8] L. Martínez, R. Andrade, E. G. Birgin, J. M. Martínez, *J. Comput. Chem.* **2009**, *30*, 2157.

[9] B. Thole, *Chem. Phys.* **1981**, *59*, 341–350.

[10] S. Y. Noskov, G. Lamoureux, B. Roux, *J. Phys. Chem. B* **2005**, *109*, 6705–6713.

[11] T. Taylor, M. Schmollngruber, C. Schröder, O. Steinhauser, *J. Chem. Phys.* **2013**, *138*.

[12] K. T. Tang, J. P. Toennies, *The Journal of Chemical Physics* **1984**, *80*, 3726–3741.

[13] S. Plimpton, *J. Comput. Phys.* **1995**, *117*, 1.

[14] J. Qian, W. A. Henderson, W. Xu, P. Bhattacharya, M. Engelhard, O. Borodin, J.-G. Zhang, *Nat. Commun.* **2015**, *6*, 6362.

[15] Y. Zhao, T. Zhou, T. Ashirov, M. E. Kazzi, C. Cancellieri, L. P. Jeurgens, J. W. Choi, A. Coskun, *Nat. Commun.* **2022**, *13*, 2575.

[16] I.-C. Yeh, G. Hummer, *J. Phys. Chem. B* **2004**, *108*, 15873.

[17] E. Quijada-Maldonado, S. van der Boogaart, J. Lijbers, G. Meindersma, A. de Haan, *J. Chem. Thermodyn.* **2012**, *51*, 51.

[18] C. A. N. de Castro, A. Lamas, X. Paredes, F. J. V Santos, M. J. V. Lourenço, T. A. Graber, *J. Chem. Eng. Data* **2024**, *69*, 2227.

[19] Y. Yin, J. Holoubek, K. Kim, A. Liu, B. Bhamwala, S. Wang, B. Lu, K. Yu, H. Gao, M. Li, G. Raghavendran, G. Cai, W. Li, P. Liu, Y. S. Meng, Z. Chen, *Angew. Chem., Int. Ed.* **2025**, *64*, e202420411.

[20] P. Zheng, X. Meng, J. Wu, Z. Liu, *Int. J. Thermophys.* **2008**, *29*, 1244.

[21] M. D. Ahmed, Z. Zhu, A. Khamzin, S. J. Paddison, A. P. Sokolov, I. Popov, *J. Phys. Chem. B* **2023**, *127*, 10411.

[22] E. J. Maginn, R. A. Messerly, D. J. Carlson, D. R. Roe, J. R. Elliot, *Liv. J. Comput. Mol. Sci.* **2019**, *1*, 6324.

[23] A. Hockmann, P. Yan, D. Diddens, I. Cekic-Laskovic, M. Schönhoff, *J. Phys. Chem. B* **2025**, *129*, 6289.

[24] A. Hockmann, F. Ackermann, D. Diddens, I. Cekic-Laskovic, M. Schönhoff, *Faraday Discuss.* **2024**, *253*, 343.
